# Quantitative Analysis of the Association Angle between T-cell Receptor Vα/Vβ Domains Reveals Important Features for Epitope Recognition

**DOI:** 10.1371/journal.pcbi.1004244

**Published:** 2015-07-17

**Authors:** Thomas Hoffmann, Angela M. Krackhardt, Iris Antes

**Affiliations:** 1 Department of Biosciences and Center for Integrated Protein Science Munich,Technische Universität München, Freising-Weihenstephan, Germany; 2 Medizinische Klinik III, Innere Medizin mit Schwerpunkt Hämatologie und Onkologie, Technische Universität München, Munich, Germany; 3 Clinical Cooperation Group, Antigen specific T cell therapy, Helmholtz Zentrum München (GmbH), German Center for Environmental Health, Munich, Germany; 4 German Cancer Consortium (DKTK), Munich, Germany; La Jolla Institute for Allergy and Immunology, UNITED STATES

## Abstract

T-cell receptors (TCR) play an important role in the adaptive immune system as they recognize pathogen- or cancer-based epitopes and thus initiate the cell-mediated immune response. Therefore there exists a growing interest in the optimization of TCRs for medical purposes like adoptive T-cell therapy. However, the molecular mechanisms behind T-cell signaling are still predominantly unknown. For small sets of TCRs it was observed that the angle between their Vα- and Vβ-domains, which bind the epitope, can vary and might be important for epitope recognition. Here we present a comprehensive, quantitative study of the variation in the Vα/Vβ interdomain-angle and its influence on epitope recognition, performing a systematic bioinformatics analysis based on a representative set of experimental TCR structures. For this purpose we developed a new, cuboid-based superpositioning method, which allows a unique, quantitative analysis of the Vα/Vβ-angles. Angle-based clustering led to six significantly different clusters. Analysis of these clusters revealed the unexpected result that the angle is predominantly influenced by the TCR-clonotype, whereas the bound epitope has only a minor influence. Furthermore we could identify a previously unknown center of rotation (CoR), which is shared by all TCRs. All TCR geometries can be obtained by rotation around this center, rendering it a new, common TCR feature with the potential of improving the accuracy of TCR structure prediction considerably. The importance of Vα/Vβ rotation for signaling was confirmed as we observed larger variances in the Vα/Vβ-angles in unbound TCRs compared to epitope-bound TCRs. Our results strongly support a two-step mechanism for TCR-epitope: First, preformation of a flexible TCR geometry in the unbound state and second, locking of the Vα/Vβ-angle in a TCR-type specific geometry upon epitope-MHC association, the latter being driven by rotation around the unique center of rotation.

## Author Summary

The recognition of antigenic peptides by cytotoxic T-cells is one of the crucial steps during the adaptive immune response. Thus a detailed understanding of this process is not only important for elucidating the mechanism behind T-cell signaling, but also for various emerging new medical applications like T-cell based immunotherapies and designed bio-therapeutics. However, despite the fast growing interest in this field, the mechanistic basis of the immune response is still largely unknown. Previous qualitative studies suggested that the T-cell receptor (TCR) Vα/Vβ-interdomain angle plays a crucial role in epitope recognition as it predetermines the relative position of its antigen-recognizing CDR1-3 loops and thus TCR specificity. In the manuscript we present a systematic bioinformatic analysis of the structural characteristics of bound and unbound TCR molecules focusing on the Vα/Vβ-angle. Our results demonstrate the importance of this angle for signaling, as several distinct Vα/Vβ-angle based structural clusters could be observed and larger angle flexibilities exist for unbound TCRs than for bound TCRs, providing quantitative proof for a two-step locking mechanism upon epitope recognition. In this context, we could identify a unique rotational point, which allows a quantitative, yet intuitive description of all observed angle variations and the structural changes upon epitope binding.

## Introduction

**Fig 1:**
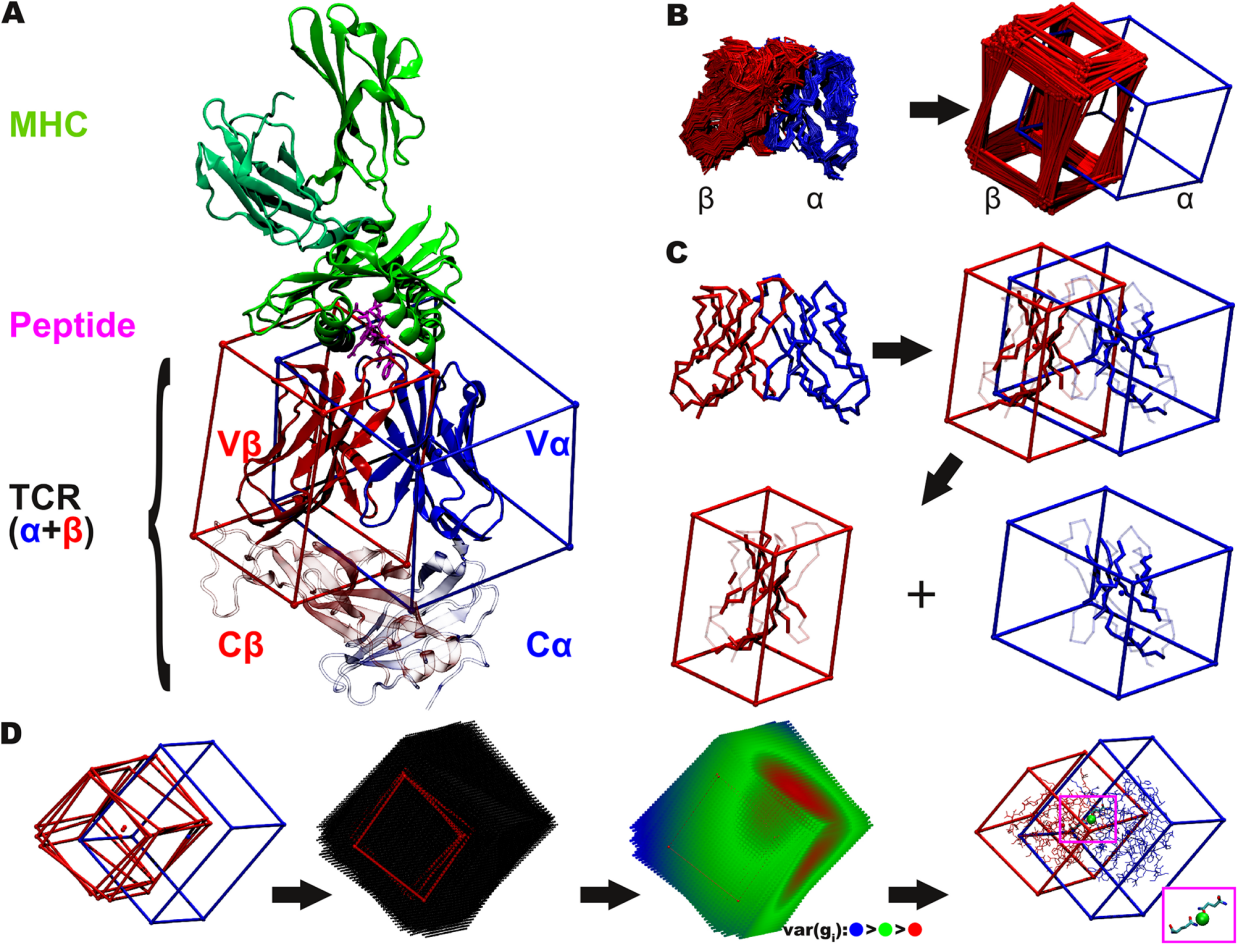
Cuboid and grid representations of the T–cell receptor geometries. (A) Localisation of the considered Vα and Vβ variable domains within the ternary TCR:pMHC complex. A TCR consists of two chains, the α and the β chain (blue and red). Each chain is partitioned into two domains, the constant domain (Cα and Cβ shown transparently) and a variable domain (Vα and Vβ, here surrounded by cuboids). The Vα and Vβ domains form the binding interface to the major histocompatibility complex (MHC) molecule (green) presenting an antigenic peptide (magenta) to the TCR. This work focuses on the variable domains. (B) Superimposition of the TCR variable domains. (i) The TCR structures were superimposed on the Vα domains leading to displaced Vβ domains. (ii) Cuboids were placed around the superimposed Vα and Vβ domains. This unified description of the different domains allows a quantitative analysis of the displacement. (C) Preparation of the cuboid placement templates. Vα (blue) and Vβ (red) domains of the structure 2bnu are used as reference structure. Both chains are surrounded with cuboids of the size of their spatial extent. Residues considered for superimposition are determined in an iterative process (unused residues are depicted transparently). These residues are used to compute the angular displacement of the Vβ domain relative to the Vα domain. (D) Center of Rotation (CoR). (i) Different geometries of (only three for clearness) β-cuboid geometries (red), superimposed on the α-cuboids (blue). (ii) Grids were fit into the β-cuboids. (iii) For each grid point *i*, the sum of pairwise distances and the variance was computed according to Formula 2. (iv) The residues at the center of rotation (CoR, green sphere) were investigated. For most of the structures, a conserved pair hydrogen bond interaction between the α and the β chain is located directly at the CoR. These hydrogen bonds are established by conserved Q residues.

**Fig 2:**
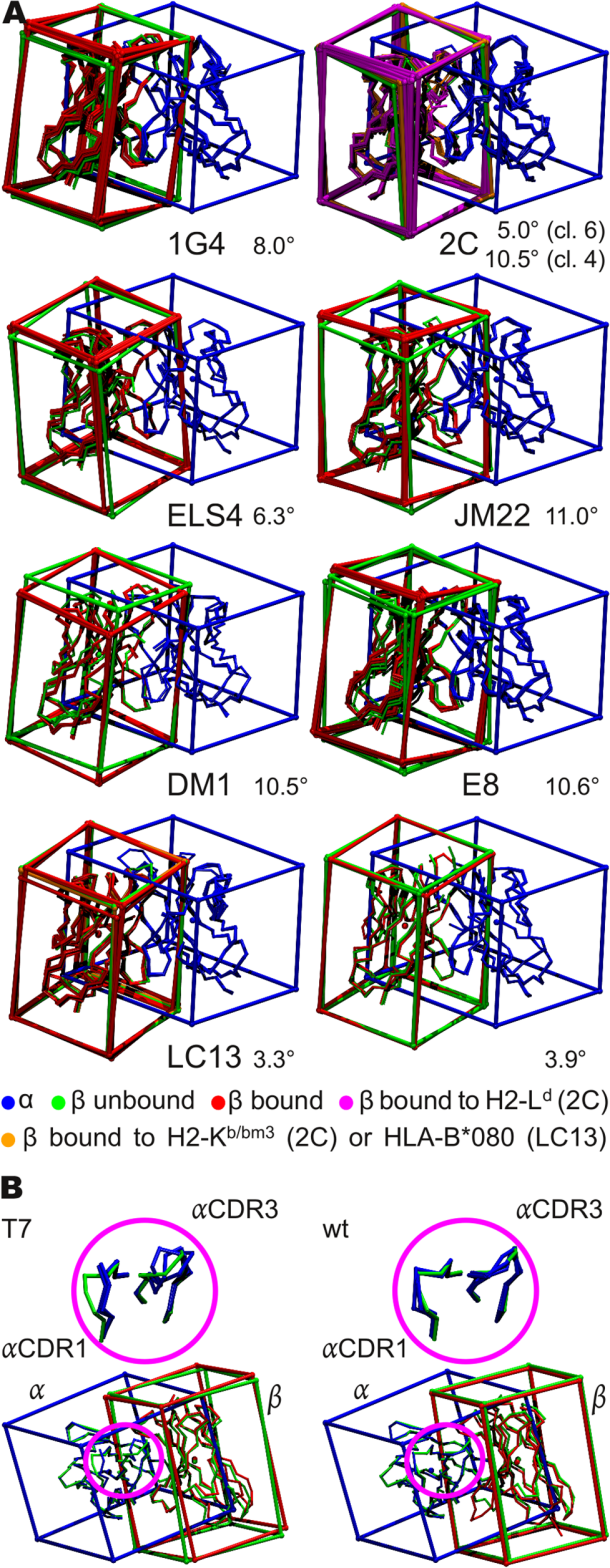
Differences in the TCR chain association geometries. (A) Differences between the bound and unbound geometries. Shown are seven different receptor types in their unbound state as well as their bound state. Notably, for the 1G4 receptor the two unbound states are derived from different crystal structures, but are very similar. For some receptors, such as the 2C receptor, crystal structures including different ligands are available. In case of the 2C TCR, wild type (wt), 2C T7 and 2C T7 mutants are shown. In case of the E8 TCR, the two unbound states are derived from the same crystal structure. (B) Different conformations of the bound 2C TCR structures and their variants. The magnifications show the different CDR1/3 conformations observed in the m67 variant structure 2e2h (green), with respect to the 2C T7 variants (right, blue, 2oi9, 3e3q, and 2e7l) and the 2C wt structures (left, blue, 1g6r, 1mwa, and 2ckb). In the lower figures both variable domains are shown together with the placed cuboids for the structures 2e2h (m67, green, left+right) in comparison to 2e7l (T7 m6, blue/red, right) and 1mwa (2C wt, blue/red, left). The αCDR3 loops of the T7 variants differ in sequence and thus in their backbone conformations, whereas the CDR1 loop conformation is the same for the T7-wt, m6, and m13, but differs for m67 (upper left magnification). In the case of the m67 variant the CDR3 and CDR1 loop conformations are consistent with the 2C wt conformations (upper right magnification).

T-cells play a major role in cell-mediated adaptive immune responses necessary for the defense against foreign invaders and transformed malignant cells. Heterodimeric T-cell receptors (TCR) recognize antigenic peptides presented on the surface of cells by major histocompatibility complex (MHC) molecules. Recognition of MHC molecules presenting foreign peptides induces TCR signaling leading to T cell expansion and specific T cell functions such as elimination of virus-infected or transformed target cells. Therefore the immune system needs to balance the subtle distinction between self-restriction and self-tolerance and responses may reach extremes from multifunctional T-cell activation to tolerance induction. Due to the complexity of the signaling process, its mechanistic details are still not well understood. Several mechanisms of signal transduction have been proposed, which can be classified into (i) aggregation-, (ii) conformational change-, and (iii) segregation-models [1–3]. These three classes are not mutually exclusive. A conformational change in the TCR associated CD3 molecule was observed to be a basic early event in the signaling cascade [4]. In this context, mechanical forces applied by the TCR domains to the associated coreceptors are a suggested explanation [5]. Recent studies showed an antigen-specific conformational change of the A-B loop of the TCR constant α (Cα) domain for at least two TCR types. However, neither the structural details of this inter-subunit communication nor its initiation mechanism are yet known [4]. In order to provide the TCRs complex functions required for the signaling process, a variety of regulatory elements are involved in the process. Among those are the conformational changes within the TCR that are triggered during the early stage binding to the peptide-MHC (pMHC) complex. TCRs structurally consist of two membrane-anchored chains (α and β chain), which form two domains with an immunoglobulin-like (IG-) fold, one constant and one variable domain (Cα, Cβ, Vα, and Vβ). The variable domains of the two chains associate to the Vα:Vβ-complex, which binds to the pMHC-complexes and thus is responsible for antigen recognition. The overall structure is Fab-fragment like and each Vα and Vβ domain consists of a framework region and three antigen-MHC specific recognition loops, the CDR1 to CDR3 loops (Fig 1*A* and Fig 2*B*).

The capability of the immune system to recognize many different pMHC complexes is achieved by a vast variety of different TCRs. The T-cell repertoire was estimated to 2.5x10^7^ for one human individual [6], and to 2x10^6^ for mice [7]. This genetic variety together with the associated conformational differences within the TCRs seem to contribute to the structural and functional plasticity of TCRs [8]. The highly variable CDR3 loops encoded by VDJ recombinations are responsible for specific peptide recognition. Conformational changes within the CDR3 region after assembly with the pMHC complex have been demonstrated to provide an adaption to distinct peptide-MHC pairs which may additionally be influenced by CD8 co-receptor binding [9]. Moreover, the presence or absence of co-receptors as well as co-stimulatory molecules can have opposite effects on distinct TCR and T-cells suggesting an additional module for regulation [10]. Recently, it has been described that TCR Vα and Vβ domains can switch among alternate conformations when binding to MHC class I or II peptide complexes. A flex point in the FGXG motif of the J element has been proposed as swivel point for adjusting the interaction of Vα and Vβ [11]. In 1997 Li *et al*. proposed the capability of TCRs to increase their plasticity by rearranging the relative orientation of the Vα/Vβ domains, analogous to several known rearrangements of the V_L_/V_H_ domains of antibodies [12]. Later works of Gagnon *et al*. reported a shift in the Vα/Vβ orientation of the A6 TCR bound to different ligands and influence of these shifts on the constant domains of the TCRs [13]. When the first structure of an A6:Tax:HLA-A2 complex was resolved, small variations in the Vα/Vβ interdomain angles could be determined [14,15]. This system was further studied with different agonistic or antagonistic peptides [16] and it was found, that different peptides induced these minor changes in the relative Vα/Vβ association geometries. Studies of the fluorination of the Tax-Peptide to increase the affinity confirmed this effect and also showed an alteration of the relative angle of the constant domains [13]. These scissoring effects were also observed for other receptors with different ligands or comparing the bound and unbound state: 2C [17], HA1.7 [18], LC13 [19], JM22 [20], DM1 [21], sc1.D9.B2 [22] and also for an invariant natural killer T cell receptor (NKT) [23].

The conformational changes were rather seen as further degree of freedom of the TCRs to adapt to the shape of their ligands [20,22]. A direct relationship between different conformational Vα/Vβ adjustments was not found [16].

In 2008, McBeth *et al*. systematically determined the Vα/Vβ interdomain angles for 35 TCR structures and concluded, that this angle is a general property of TCRs, which expands the repertoire of specificity [22]. Similarly, two recently published studies of Dunbar *et al*. investigate the interdomain geometries of antibodies [24] and compare them to the geometries of a non-redundant set of 39 structures [25]. The structure of TCRs is similar to Fab-fragments of antibodies [26], whereby the antibody V_L_/V_H_ correspond to the TCR Vα/Vβ domains. In early and recent studies of antibody structures a rearrangement of the V_L_/V_H_ upon ligand binding was considered and later confirmed [27–39]. Knowledge about TCR chain interactions might not only be important for the understanding of different TCR functions but may additionally provide information for reliable prediction of chain pairing. This is particularly interesting in T-cell based immunotherapy in which TCRs are considered as therapeutic tools for viral diseases and various cancers. For this purpose T cells redirected after genetic transfer of TCR chains with defined specificity are applied [40]. Understanding of TCR chain assembly is highly important in this regard as incorrect binding of introduced TCR chains with an endogenous TCR α and β chain may result in severe morbidity [41].

In the field of rational TCR engineering and optimization, homology modeling of these receptors gained in importance. Michielin *et al*. early created a homology model of the T1 TCR [42] using the MODELLER tool [43]. Later, other distinct TCR:pMHC models were investigated using more elaborate techniques including molecular dynamics (MD), computational alanine scan, or free energy calculations to study the influence of single mutations in the TCR or in the ligand, or to study differences of similar systems [44–54], and since recently, the automated modeling approach TCRep 3D is available to predict arbitrary TCR:pMHCI complex structures [55]. Recently Knapp *et al*. applied the ABangle methodology to a broad range of MD simulations of the LC13 TCR bound to 172 different ligands [24,56]. However, none of the previous modeling approaches explicitly includes any features concerning potential alterations in the Vα/Vβ interdomain angles, thus the presented structural analysis can help to improve the performance of the existing TCR modeling approaches.

In this work we perform a systematic, quantitative analysis of the Vα/Vβ interdomain angles in experimental TCR structures. For this purpose we developed a new structure-based method, which allows a systematic and very accurate quantitative comparison of the differences in the Vα/Vβ interdomain angles and introduces a new distance measure for clustering leading to a more accurate structural alignment of the TCRs than the approaches used in previous studies. The determination of TCR interdomain geometries is complicated by the fact that structural data is only available for a small subset of the vast variety of TCRs and that the TCRs for which structural data is available differ considerably in their loop structure and chain length, rendering the location of common conserved structural elements difficult. To solve these complications our method transfers all TCR variable domains into a unified geometric scaffold and performs a systematic analysis of the TCR structure geometries for 85 representative structures with respect to their Vα/Vβ interdomain geometries and interactions.

## Results

To analyze relative positions of the Vα and Vβ domains of all bound and unbound TCR structures in the dataset (Table 1, S1 Table and S2 Table) we introduced a new methodology which assigns uniquely defined cuboid-based frames to the individual Vα and Vβ domains of the TCRs and thus allows an unambiguous analysis of their relative geometries (for details see Methods). Based on this method we first examined the relative positions of the two domains with respect to each other and then performed a throughout analysis of the structural basis of the obtained observations.

**Table 1:** All TCR structures used for the analysis.

Name	S^a^	BS^b^	PDB	Name	S^a^	BS^b^	PDB
1G4	h	u	2bnu	LC13	h	1	3kps
1G4	h	1	2bnq	MEL5	h	1	3hg1
1G4	h	1	2bnr	OB.1A12	h	2	2wbj
1G4 AV-wt	h	1	2f54	OB.1A12	h	2	1ymm
1G4 c5c1	h	u	2pyf	RA14	h	1	3gsn
1G4 c5c1	h	1	2pye	SB27	h	1	2ak4
1G4 c49c50	h	1	2f53	SB27[K16Dα]	h	1	3kxf
1G4 c58c62	h	1	2p5w	TCR MS2-3C8	h	2	3o6f
1G4 c58c61	h	1	2p5e	TK3 wt	h	1	3mv7
3A6	h	2	1zgl	TK3 Q55H	h	1	3mv8
A6	h	1	2gj6	TK3 Q55A	h	1	3mv9
A6	h	1	1qsf	1934,4	m	2	2pxy
A6	h	1	1qse	1F1E8	m	u	3mff
A6	h	1	3d3v	226 TCR	m	2	3qiu
A6	h	1	3d39	226 TCR	m	2	3qiw
A6	h	1	1qrn	2B4	m	1	3qib
A6	h	1	1ao7	2B4	m	u	3qjf
A6	h	1	3h9s	2C	m	u	1tcr
A6	h	1	3pwp	2C	m	1	1g6r
AS01	h	1	3o4l	2C	m	1	1mwa
B7	h	1	1bd2	2C	m	1	2ckb
cf34	h	1	3ffc	2C T7	m	s,(2) ^c^	2icw
DM1	h	1	3dxa	2C [T7-wt-s] ^d^	m	1	2oi9
DM1	h	u	3dx9	2C m13 [T7-s] ^d^	m	1	3e3q
E8	h	2	2ian	2C m6 [T7-s]^d^	m	1	2e7l
E8	h	2	2iam	2C m67 [T7-s] ^d^	m	1	3e2h
E8	h	u	2ial	2W20	m	2	3c6l
ELS4	h	1	2nx5	5c.c7	m	u	3qjh
ELS4	h	u	2nw2	AHIII12.2	m	1	2uwe
HA1.7	h	2	1fyt	AHIII12.2	m	1	2jcc
HA1.7	h	2	1j8h	AHIII12.2	m	1	1lp9
Hy.1B1	h	1	3pl6	B3K506	m	2	3c5z
JM22	h	1	2vlj	BM3.3	m	1	1nam
JM22	h	1	2vlk	BM3.3	m	1	1fo0
JM22	h	1	1oga	BM3.3	m	1	2ol3
JM22	h	u	2vlm	cl19	m	2	2z31
JM22	h	s,(2)^c^	2xn9	D10	m	2	1d9k
JM22	h	s	2xna	KB5-C20	m	1	1kj2
JM22 [S99βA]	h	1	2vlr	N15	m	u,a	1nfd
KK50.4	h	1	2esv	TCR 21.30	m	2	3mbe
LC13	h	1	1mi5	TCR172.10	m	2	1u3h
LC13	h	u	1kgc	YAe62	m	2	3c60
LC13	h	1	3kpr				

^a)^ Species: h = *homo sapiens*, m = *mus musculus*.

^b)^ Bound state: u = unbound, 1 = MHCI, 2 = MHCII, s = superantigen. More detailed information is available in S1 Table.

^c)^ Structure 2xn9 and 2icw are not considered as MHC II bound TCRs, since the TCRs only contact the super-antigens.

^d)^ WT with solubility mutations acc. to ref. [63]. More detailed information is available in S2 Table.

### Cluster analysis of the TCR Vα/Vβ association angles

For the analysis of the relative Vα and Vβ domain geometries we superposed the Vα domain of these structures and investigated the differences in the position of the corresponding Vβ domains using their assigned cuboid frames. For this purpose a conserved framework region was identified in both TCR chains and cuboids were placed around each variable domain centered on the framework region (Fig 1*B–1D*). Afterwards the relative Euler angles of the Vβ cuboids were measured with respect to the superposed Vα domains.

**Fig 3:**
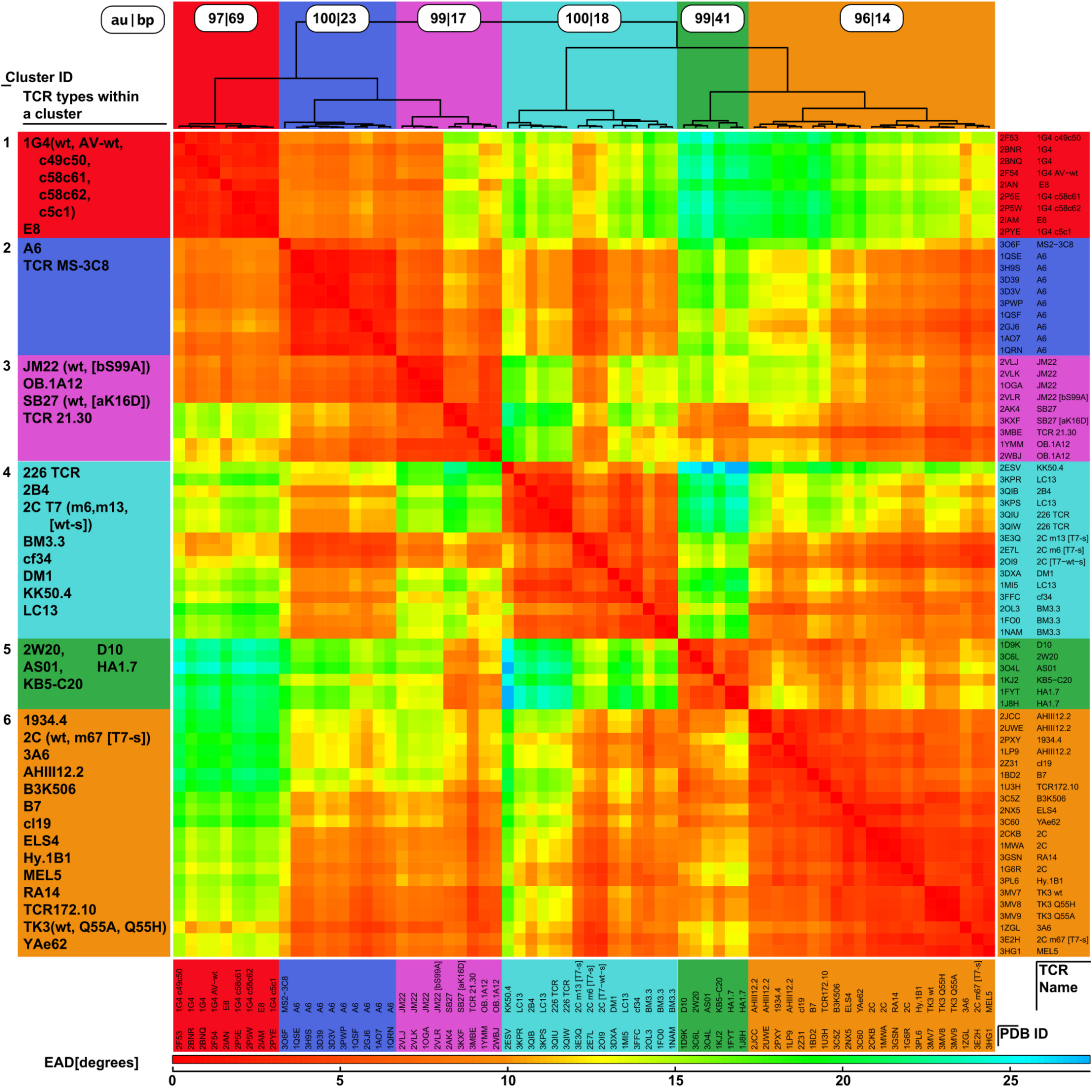
Geometry clusters of pMHC bound TCRs. Pairwise Euler-angle distances (EAD) were determined for all pMHC-bound TCR structures according to Formula 1. The distance matrix was hierarchically clustered using the Ward update formula. We identified six significant clusters, using a bootstrapping approach [58]. Notably, in most of the cases, TCRs of the same type occur in the same cluster. Upper panel: Clustering dendrogram with bootstrapping results (au = approximately unbiased, bp = bootstrapping probability). Left panel: TCR types occurring within a cluster. Right/lower panel: PDB identifiers and corresponding TCR names. Central panel: Pairwise Euler-angle distances (EAD). The color key is provided in the bottom of the figure.

The analysis showed that the relative positions of the Vα and Vβ domains of the TCRs differ considerably with respect to each other (Fig 3), which is consistent with former qualitative studies on small subsets or individual TCRs [22]. In Fig 1*B* it can be observed that if the central β-sheets of the Vα domain are superposed very well, the backbone positions of the corresponding Vβ domains differ significantly featuring interdomain Euler-angle distances *d*_*E*_ up to 30° (see Methods). Therefore, the two TCR binding domains can adopt different orientations (Fig 1*B*) with respect to each other. To analyze these differences in more detail we clustered all superposed (i) MHC-bound structures (Fig 3 and S1 Fig) as well as (ii) all MHC bound and unbound structures (S2 Fig and S3 Fig) according to their angular deviations in the Vβ domains with respect to the corresponding Vα domain using the Ward clustering algorithm [57]. Afterwards we performed a bootstrapping analysis (Fig 3) and identified six clusters with a significance greater than 95% [58]. Nearly all structures of TCRs of the same type from which different X-ray structures exist were placed in the same cluster (93% of the TCR types with more than one MHC bound crystal structure; except 2C TCR). This shows that the observed phenomenon is not caused by the variation of the crystallographic conditions and that the clustering is robust, describing a phenomenon which is caused by biological differences within different types of TCRs.

### Structural analysis of the TCR cluster geometries

After the angle-based cluster analysis of the superposed structures we analyzed the structural features leading to the different interdomain geometries observed. For this purpose we used a grid-based analysis of the superimposed cuboid structures (Fig 1*D*, for details see Methods). This analysis showed that all TCR structures share an area, which is invariant towards rotation and translation of the TCR variable domains. At the center of this region a rotation point (Center of Rotation, CoR) can be identified, which exists in all TCR structures. The core region around this CoR is situated at the center between the two Vα and Vβ domains (Fig 1*D*). Notably, the average CoR position (x = 27.768Å, y = 36.783Å, z = 55.723Å) with respect to the reference coordinate system (2bnu) is located directly between or close to a twofold hydrogen bond between two conserved residues (Q for most of the structures), one from each chain (Fig 1*D*, magenta box). These hydrogen bonds connecting the two chains are known to be conserved through all TCRs [59]. As similar structural constraints were observed for antibodies [59,60], these features (CoR stabilized by conserved H-bonds) seem to be characteristic for Fab-fragment like domains in general.

To investigate the conservation of these two residues, we performed a sequence-based analysis with the sequences of all currently known functional variable αβ TCR gene segments as found in the database IMGT/GENE-DB [61]. In total 342 α chain and 164 β chain sequences were analyzed (six of 348 α sequences were incomplete). This analysis shows, that in contrast to antibodies, different residues can be found at the CoR position (Table 2 and S3 Table). Table 2 provides the absolute number of the observed amino acids at the CoR position separately for the known α- and β-alleles. The investigated CoR position corresponds to sequence position 44 in the IMGT unique numbering [62] scheme for both, the Vα and the Vβ domains. In case of the α chain the amino acids Q, H, R, K, L, W, and E can be observed, whereas in case of the β chain Q is overrepresented, but is occasionally replaced by R and K. The lower amount of different residues found in the β chain alleles might be a statistical artifact, since for the α chain about twice as many sequences are known than for the β chain.

**Table 2:** Conservation at the CoR position.

AA^a^	Freq. α [%]		Freq. β [%]	
Q	89.2	(305)	98.2	(161)
R	26.0	(9)	1.2	(2)
E	0.3	(1)		
H	5.0	(17)		
W	0.6	(2)		
K	1.8	(6)	0.6	(1)
L	0.6	(2)		

Relative (and absolute) Frequency of the AA at the α or β CoR position, based on an multiple sequence alignments of all functional variable TCR gene segments alleles of the α (342 sequences) or β (164 sequences) locus obtained from the IMGT/Gene-DB [61].

^a)^ amino acid type

**Fig 4:**
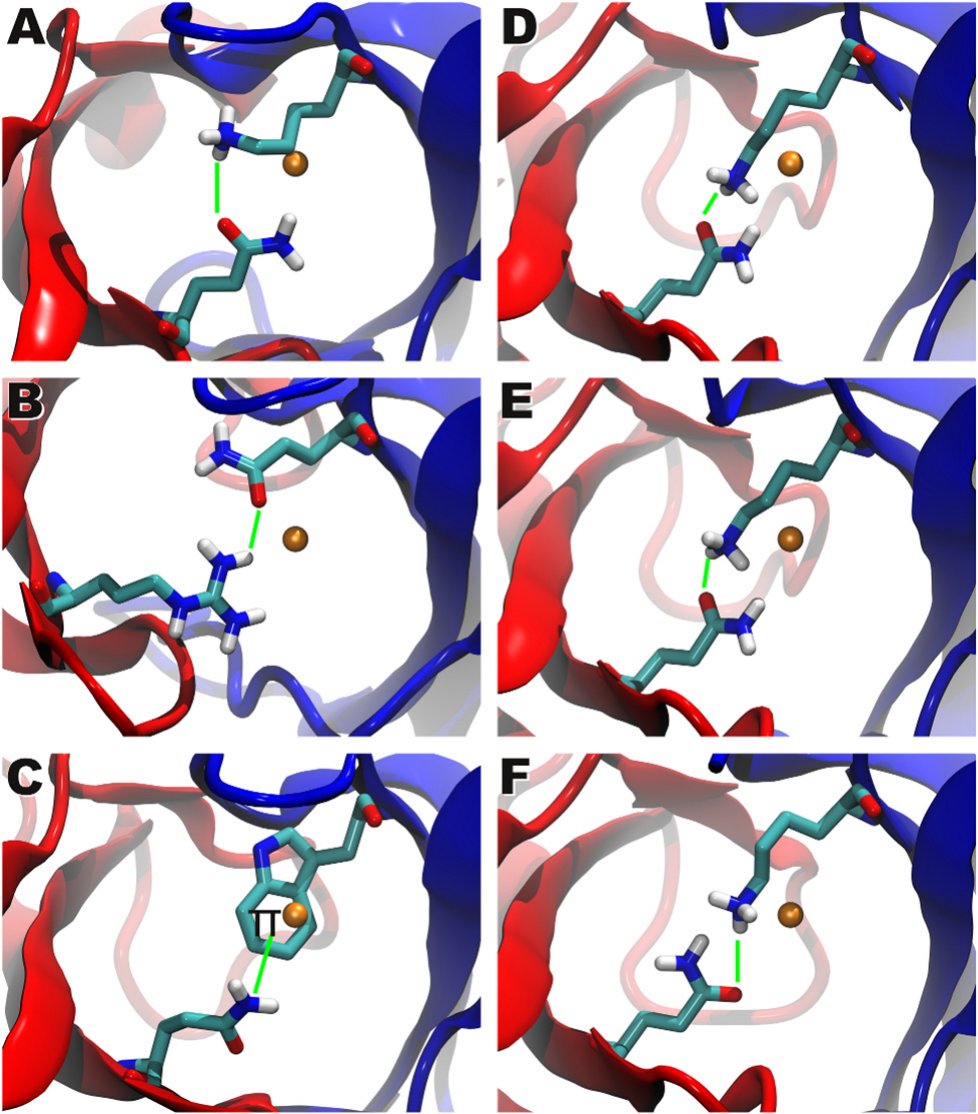
Exceptional structural examples of the center of rotation. Region around the Center of Rotation (CoR), the Vα domain is shown in blue and the Vβ domain in red. Hydrogen atoms were added for the end-groups of the interacting amino acids. The average center of rotation is drawn as an orange sphere and the interacting residues are shown in licorice representation. CoR stabilizing interactions are drawn as a green line. For these six structures the highly conserved Q-Q interaction between the α and the β chains is replaced by the following residues (shown in licorice style): (A, D-F) αK (PDB-IDs: 1bd2, 3qiu, 3qiw, 3qjh), (B) βR (PDB-ID: 2esv), (C) αW (PDB-ID: 3gsn).

Structural investigation of the interaction pattern of these alternative residues observed at position 44 showed that all of them can form strong interactions with their interacting partner residue in the complementary chain and thus compensate for the lost hydrogen bonds of the Q-Q interaction (see Fig 4): In some cases the Q residue of the α chain is replaced by an apolar W or L residue. In the case of W the Π-system of its indolyl group forms strong interactions with the Q residue from the opposite chain. In most cases Q is replaced by R or K and therefore the formation of the interchain hydrogen bonds can still be observed, as shown in Fig 4. As no structures are available for the replacement of Q by E or H no structural analysis is possible for these mutations. The same holds for the K mutant in the β chain, as in all available structures the conserved position in the β chain is occupied by Q except for the TCR KK50.4 (structure 2esv), where Q is replaced by R (Fig 4*B*). In this structure the side chain oxygen atom of the Q residue of the α chain forms a hydrogen bond with the guanidine group of the R residue. Compared to structures with Q-Q interactions, the Q residue is slightly displaced towards a neighboring loop, due to the size of the interacting R residue. This displacement allows a further interaction of the Q residue with a backbone carbonyl-oxygen of the neighboring loop. The α chain offers more diversity: K residues are found at the α-CoR position in the TCRs B7 (PDB ID 1bd2), 226 TCR (PDB IDs 3qiu, 3qiw), and 5c.c7 (PDB IDs 3qjh) (Fig 4*A*, 4*D–4F*). The rare W residue at the α-CoR position can be observed in the RA14 TCR (PDB ID: 3gsn, Fig 4*C*). In the β chain of the B7 TCR (1bd2) the CoR position is occupied by a conserved Q residue, the side chain oxygen is directed towards the side chain nitrogen atom of the K residue at the CoR position of the α chain. The distance between the two atoms is 3.59 Å. The K residue is drawn towards a neighboring loop, such that the amino group can also interact with an oxygen atom of the backbone of the loop (distance N-O: 2.89 Å). This additional interaction stabilizes the conformation of the K residue. For both “226 TCR” structures (3qui and 3qiw) very similar conformations of K- and the Q-residue can be observed: the side chain nitrogen atom of the K of the α chain is directed towards the side chain oxygen of the Q (distance in 3qui: 2.39 Å; 2.60 Å for 3qiw). In the 5c.c7 structure (3qjh) the atomic coordinates of the two observed Q and K residues are very similar compared to the two “226 TCR” structures. However, the oxygen atom and the nitrogen atom of the Q residue are swapped in one of the BUs, so that the K nitrogen is directed towards the nitrogen atom of the Q. In the RA14 TCR (PDB ID 3gsn), where W occurs at the CoR position, the conformation of the W residue is stabilized by a hydrogen bond (distance 2.15 Å) between the nitrogen NE1 and a backbone carbonyl-oxygen of the neighboring loop of the α chain. The W residue flanks the hydrophobic core of the TCR. The Q residue of the β chain pushes towards the solvent, due to the size of the W residue. For E and H we also expect the formation of hydrogen bonds with the β chain, however, no structure exist of this case yet.

Overall, the existence of the conserved CoR in such close proximity to the conserved αQ-βQ interactions confirms the hypothesis of a rotation-driven mechanism of α:β-association leading to the differences in the association angles of the Vα and Vβ domains. However, due to the low amount of mutated sequences available it was not possible to investigate the influence of the different amino acids occupying the conserved position 44 on the TCR interdomain geometry and the TCR specificity in a comprehensive manner. In general the above observations suggest an association mechanism of the Vα and Vβ domains in which the hydrogen bond interaction between the conserved residues are formed first and afterwards the domains arrange each other around this pivot point, adopting different relative association angles.

### Detailed analysis of specific TCR:pMHC structures

Next we performed a more detailed functional and structural analysis of the clustering results. For several different TCR types (2C, A6, 1G4, JM22, BM3.3, AHIII12.2, TK3) there exist more than one structure within the analyzed dataset in which the TCR is either bound to different MHC alleles and/or different peptides or different variants of the same TCR were crystallized.

These structures differ in several features: i) mutations in the TCR framework (S2 Table) or CDR-loop regions, ii) different presenting MHC molecules (including different alleles, single point mutations, and different MHC classes), and iii) different peptides presented to the TCR (including single point mutations). Furthermore, the data set includes the two similar TCRs “2B4” and “226”, which both share the gene loci for their variable segments of their α chains as well as their β chains, but differ in the loci for the joining segments.

Based on our cluster analysis we can distinguish between *major* and *minor* angular differences. According to our definition minor differences between two TCR structures occur if both structures can be found within the same cluster. Major angular differences between two TCR structures can be found for structures assigned to two distinct clusters.

Analyzing the clustering behavior of the different structures available for the same TCR types and their variants we observed the interesting results that for all except one TCR type (2C) all structures belonging to the same TCR type are located within one cluster (Fig 3). Thus, we investigated this phenomenon in more detail. In this section we briefly summarize the main results and refer the interested reader to a more detailed description in the supporting material (S1 Text).

Detailed analysis of the different structures available for the TCR types A6, 1G4, JM22, BM3.3, AHIII12.2, and TK3 shows that neither mutations within the TCRs nor the binding to different peptidic ligands of varying immunogenicity (e.g. A6 [13–16], S1 Table) or MHC alleles lead to major angular differences. However, minor angular differences are frequently observed within the individual sets (see Supporting Material).

In contrary, the 2C TCR can be found in two different angular clusters (Fig 3, Table 3). This is in agreement with the original publications, which show that depending on the pMHC bound, the 2C TCR can adopt two distinct docking orientations [63], but that on the other hand mutations in the CDR3 loop of the TCR do not lead to significant changes in its orientation with respect to the pMHC ligand, if the same pMHC is bound [64]. Regarding the bound pMHC alleles in our two clusters, we find that all TCRs bound to the MHC molecule H2-K1^b^ associate in cluster 6, whereas all TCRs bound to the MHC molecule H2-L^d^ are located in cluster 4, except for the m67 variant, which is bound to H2-L^d^, but located in cluster 6. As the reason for this unusual behavior of the m67 variant it was found that its mutation of the αCDR3 loop sterically enforces a conformation of the neighboring αCDR1 loop (binding the MHC molecule), which leads to a shift between the Vα domain and thus to different interdomain angles, closer to cluster 6 (Fig 2). The same conformation shift is also observed in the experimental publication, but as the docking orientation of the Vβ-domain on the MHC is retained and only the relative Vα loops shift, no significant changes are observed with respect to the overall docking orientation [64]. Due to this surprising result we had a closer look at the structures and discovered that actually two subtypes of the 2C TCR were crystalized: the wild type (wt) and the 2C T7 TCR, which differ in the framework region (S2 Table). The wt 2C TCRs are all bound to the MHC molecule H2-K1^b^ and associate in cluster 6, whereas the T7 TCRs are all bound to the MHC molecule H2-L^d^ and belong to cluster 4, except the m67 variant. Therefore the two TCR structures compared in [63] actually belong to two different variants and thus the results and conclusions of that publication, namely that the different docking orientations are solely caused by the different pMHC ligand bound, need to be regarded with caution. Unfortunately, as for both variants only bound structures to the same pMHC are available and no “cross” TCR type ⇔ pMHC allele structures, it can not clearly be distinguished if the differences in the docking orientation and Vα/Vβ angles are caused by the framework mutations or the different pMHCs bound. However, regarding the above discussed 2C T7 m67 TCR, which is bound to H2-L^d^ and has the T7 framework mutations, but still adopts an angular conformation closer to the 2C-wt:H2-K1^b^ (Table 3, underlined) structures belonging to cluster 6 (Fig 2*B*) [64], it seems that neither of the above features (framework mutation or pMHC allele bound) seems to induce unsurmountable restrictions on the final TCR conformation. As the m67 variant is the only T7 variant, which adopts the 2C wt Vα/Vβ angle, the induced changes in its αCDR1 conformation, which are not present in the other T7 variants (Fig 2*B*), seem to play a crucial role for the Vα/Vβ association angle, whereas the αCDR3 conformation influences the angle only indirectly.

**Table 3:** Pairwise Euler Angle Distances [°] of the bound and free 2C TCR variants.

PDB	C^a^	S^b^	L^c^	1tcr	2ckb	1mwa	1g6r	3e2h	2e7l	2oi9	3e3q
1tcr	-	wt	U	0,0	3,4	4,1	5,9	6,8	9,9	9,5	12,1
2ckb	6	wt	*K*E	3,4	**0,0**	**0,8**	**2,9**	**3,5**	6,6	6,2	8,8
1mwa	6	wt	*K*E	4,1	**0,8**	**0,0**	**2,1**	**2,8**	5,9	5,4	8,0
1g6r	6	wt	*K*S	5,9	**2,9**	**2,1**	**0,0**	**2,0**	4,3	3,8	6,3
3e2h	6	m67	*L*Q	6,8	**3,5**	**2,8**	**2,0**	**0,0**	3,5	2,8	5,5
2e7l	4	m6	*L*Q	9,9	6,6	5,9	4,3	3,5	**0,0**	**2,0**	**2,3**
2oi9	4	[T7-wt-s]	*L*Q^d^	9,5	6,2	5,4	3,8	2,8	**2,0**	**0,0**	**3,0**
2e3q	4	m13	*L*Q	12,1	8,8	8,0	6,3	5,5	**2,3**	**3,0**	**0,0**

The structures were superimposed to the α variable domains. All Euler angle distances are given in degrees in respect to averaged geometries of all biological units. Unlike other 2C T7 variants (m6, T7-wt-s, m13) the m67 variant (underlined) affiliates to cluster (C) 6 occupied by the 2C wt TCRs bound to a different ligand. The two clusters are emphasized by bold typesetting.

^a)^ Cluster affiliation.

^b)^ Subtypes.

^c)^ Ligands: U = unbound, *K*E = H2-K1^b^+EQYKFYSV, *K*S = H2-K1^b^+SIYRYYG, *L*Q = H2-L^d^+QLSPFPFDL. The ligand main type (MHC) is indicated by the first letter in italics.

^d)^ MHC mutation: (F9Y)(V12T)(I23T).

These results indicate that the 2C TCR can in principle adopt two distinct conformations, which can be modulated by framework as well as the CDR mutations and presumably also its binding partner.

This is in agreement with previous qualitative observations about the overall TCR:pMHC association angle, stating that this angle is mainly dependent on the nature of the MHC allele and the TCR type rather than the antigenic peptide molecule [22,63–65]. As the CDR1 and CDR2 loops are interacting with the MHC molecule and the CDR3 loop predominantly with the bound peptide, the observed CDR1 dependent structural changes are in agreement with these former studies and might be a complementary feature to the CDR3-peptide binding in the process of TCR signaling. However, as these observations are based on one TCR only, these conclusions should be taken with caution.

### Comparison of bound and unbound TCRs

To further investigate the influence of the pMHC complex on the overall TCR structure we compared the structural features of unbound and bound TCR structures of the same type (Fig 2*A*, S2 Fig and S3 Fig). We observed that in most of the cases the orientations of the unbound TCRs slightly differ from the bound TCRs. The seven TCR types 1G4, 2C, DM1, ELS4, JM22, 2B4, and LC13 can be found in the unbound state as well as in the MHC bound state in our data set–TCRs bound to superantigens are not considered. Only in the case of the 2B4 TCRs and the LC13 TCRs both states associate in the same clusters. In the other cases, the angular deviation of the unbound TCRs is between 5° to 11°, leading to an association to a different cluster than the bound variants. Comparing all examined structures of bound and unbound TCRs it can be observed that the differences in the Vβ domain orientations are considerably larger for the unbound TCRs (S3 Fig).

In Fig 2*A* the differences between the bound and the unbound structures are illustrated for several TCR types. The repertoire of analyzed 1G4 TCRs contains nine structures of wt TCRs and mutants. Two different structures are available in the unbound state: (i) The structure 2bnu is the wt and (ii) 2pyf is the variant c5c1, which differs from the wt in the αCDR3-, βCDR2-, βCDR3-loops, and in three positions of the framework region [66, 67]. The subset of bound 1G4 TCRs contains wt TCRs (2bnq and 2bnr), the variant wt-AV (2f54, contains solubility mutations in the framework region [68]), and variants, which contain mutations in the framework region and the αCDR2-, αCDR3-, βCDR2-, βCDR3 loops: c5c1 (2pye), c49c50 (2f53), c58c62 (2p5w), and c58c61 (2p5e)–S2 Table lists the mutations in detail. All ligands of the bound 1G4 TCR structures are the MHC molecule HLA-A*0201 presenting the peptide SLLMWITQ**C** (except 2bnq: SLLMWITQ**V**).

Notably, the two unbound orientations differ only by 2.5°, but have an average distance of 8.0° to the bound structures (Table 4). On the other hand, all bound 1G4 TCR structures are very similar (2.1°, var = 0.7°). This indicates a shift in the relative orientation of the two domains upon binding of the TCR to the peptide-MHC complex. Both unbound structures associate in cluster 2, differently to the bound 1G4 TCRs, which are found in cluster 1.

**Table 4:** Pairwise Euler Angle Distances [°] of the bound and free 1G4 TCR variants.

PDB	S^a^	L^b^	2bnq	2bnr	2f53	2f54	2p5e	2pye	2p5w	2pyf	2bnu
2bnq	W	v	0,0	1,3	1,5	1,2	2,2	2,4	2,5	7,2	7,9
2bnr	W	c	**1,3**	0,0	2,7	2,2	2,5	2,2	2,9	6,1	6,7
2f53	B	c	**1,5**	**2,7**	0,0	2,1	1,7	2,5	1,8	8,7	9,3
2f54	V	c	**1,2**	**2,2**	**2,1**	0,0	3,2	3,6	3,5	7,3	8,4
2p5e	D	c	**2,2**	**2,5**	**1,7**	**3,2**	0,0	1,0	0,4	8,5	8,7
2pye	A	c	**2,4**	**2,2**	**2,5**	**3,6**	**1,0**	0,0	1,2	7,8	7,8
2p5w	C	c	**2,5**	**2,9**	**1,8**	**3,5**	**0,4**	**1,2**	0,0	8,8	9,0
2pyf	A	u	7,2	6,1	8,7	7,3	8,5	7,8	8,8	0,0	2,5
2bnu	W	u	7,9	6,7	9,3	8,4	8,7	7,8	9,0	2,5	0,0

The structures were superimposed to the α variable domains. All Euler angle distances given in degrees. Averaged angle distances: inter unbound: 2.5°, Inter bound (bold): 2.1°, bound vs. unbound (underlined): 8.0°.

^a)^ Subtypes: W = wild type, V = AV-wt, A = c5c1, B = c49c50, C = c58c62, D = C58c61

^b)^ Ligands: u = unbound, v = SLLMWITQV+HLA-A*0201, c = SLLMWITQC+HLA-A*0201.

For both, the DM1 and the JM22 types, an angular deviation between the bound and the unbound state of 10.5/11° can be observed. The JM22 TCR was reported to reveal a considerably greater scissoring motion than other TCRs [20], which is consistent with our findings. The unbound JM22 TCR (2vlm) can be found in cluster 6, whereas the bound JM22 TCR structures are located in cluster 2. The bound (3dxa) or unbound (3dx9) DM1 TCRs can be found in cluster 4, respectively cluster 2. The unbound E8 (wt) structure (2ial) associates to cluster 1 and differs by 10.6° from the bound (wt) variants (2ian, 2iam), which associate to cluster 3. The bound variant of ELS4 (2nx5) is located in cluster 6 and differs to the unbound variant (2nw2, cluster 4) by 6.3°.

In the case of the 2C TCR, the unbound wt (1tcr) associates with cluster 6, which contains the bound 2C wt structures and the exceptional bound 2C T7 m67 variant (see above). The average angular distance of the unbound wt to these bound structures is 5.0° whereas it’s distance to the bound 2C T7 variants in cluster 4 is 10.5°. In contrast, for the LC13 and the 2B4 TCRs the angular difference between the bound and the unbound structure is low (3.3° and 3.9°, cluster 4).

Thus by including the unbound TCRs into the clustering process (S1 Fig), a tendency towards smaller significant clusters can be observed. This means, that the pMHC-ligand stabilizes the TCR variable domain geometries in a favored position. The TCRs’ ability to adopt multiple geometries might play an important role in the signal transduction and the loss of flexibility upon pMHC binding might induce an initial event in the signaling cascade.

Another interesting point is that structures from human and mouse are found in the same clusters, no differences were observed in their clustering behavior.

## Discussion

We performed a comprehensive quantitative analysis of the structural features of T-cell receptors in their bound and unbound states. For this purpose, we introduced a new cuboid-based method, which allowed us to obtain a unique quantitative measure for the Vα/Vβ association angles and thus the previously observed rotation between the two TCR domains. As our method is based on highly conserved framework residues and ignores the loop regions it can be applied to all possible chain combinations and we performed a detailed analysis based on a representative set of all currently available TCR structures in the PDB Database.

Differences in the TCR Vα/Vβ association angles were first recognized for the A6:Tax:HLA-A2 complex by Ding *et al*. [16]. Since then the same phenomenon was also observed for other TCR clonotypes by several groups [17–21,23]. The first more comprehensive analysis of the angular space of the TCR Vα/Vβ association was performed by McBeth *et al*. [22], who analyzed 38 TCR structures (biological units), including unbound TCRs, MHC I- or MHC II-bound TCRs, and three NKT TCRs. The analysis was based on three angles: two angles were defined as the pitch of a pseudodyad axis and a third angle described the rotation around this axis when superimposing the two variable domains.

The results of the study of McBeth showed that different TCRs adopt a broad range of orientations and that the orientation of TCRs of the same type in the bound and unbound states can differ. Furthermore, the authors observed angular differences between TCRs differing only in a few amino acids, concluding that the variation of the interdomain angle potentially has an effect on the TCRs specificity or polyspecificity [22].

The pseudodyad-based method used by McBeth *et al*. is a classical approach of crystallographers to determine the relative orientation of antibody V domains or to determine the antibody elbow angle in Fab fragments. The computation of the pseudodyad-axis is achieved by superimposing of the Vα onto the Vβ domain. The drawback of this approach is that the precision of this process depends on the similarity of the two domains and it can be expected that the cross-chain similarity of the variable domains is lower than the similarity between two variable domains of the same chain type (either Vα or Vβ). Thus, two variable domains of the same chain type can be structurally aligned more precisely than superimposing similar cross-chain domains. Due to these limitations we developed a new method for superpositioning, which allows a unique definition of the interdomain rotational angle by superimposing domains of the same type using structurally highly conserved regions for the superimpositioning process. Our method describes the orientation of the Vβ domain relative to the Vα domain by a unified cuboid instead of a pseudodyad-axis as used by McBeth. The cuboid-based description provides several benefits. First, only one angle is necessary to describe the interdomain rotation, which is not only intuitively accessible, but also forms the simplest description of the phenomenon and allows a straightforward bioinformatics structural analysis. Second, the Euler angle distance can be computed between cuboids, which can be used as a measure for clustering. Third, cuboid geometry combinations can be used as a template for an arbitrary cross type chain assembly in a modeling process.

Since 2008 the number of TCR structures available in the PDB increased considerably. Therefore, we were also able to base our analysis on a much broader data set. The data set of McBeth included 18 non-NKT and 3 NKT structures (38 BUs), whereas our set contains 37 different non-NKT TCR types (mutants not counted, 136 BUs). In both studies free, MHC I bound, and MHC II bound TCRs originating from human or mouse were studied. However, the recent data allowed us to compare additional TCRs in bound and unbound state (*e*.*g*. DM1, JM22, LC13, E8, 2C, 1G4). For other TCRs the new dataset contains structures with additional different pMHC ligands (*e*.*g*. A6, SB27, 1G4, 2C, LC13, Ob.1A1).

Recently another study was published by Dunbar *et al*. [25], which also analyzes the TCR Vα/Vβ interdomain angle. However, the focus of that study is on the comparison of TCR and antibody geometries, because of its importance in the field of rational design of TCR-like antibodies. This is quite different from our goal of a systematic comparison of the interdomain angle variations within different TCR structures depending on their surrounding and thus the publication of Dunbar *et al*. sheds light on an important, yet complementary aspect of TCR architecture. Due to its different focus, the study also differs in the methodology applied as well as the data set used and the results discussed. Regarding the data set Dunbar *et al*. examine a smaller set of 39 structures, which does not contain TCR type-based redundant structures to avoid statistical bias in the comparison with the antibodies. In contrast, the inclusion of different structures of the same TCR is a desired feature of our data set as our analysis focuses on the differences between the available TCR structures in dependence of their binding state and partners. However, in contrast to Dunbar’s study, we excluded NKT receptors (binding CD1d ligands) as well as structures containing superantigens as they show a different binding behavior and function and thus are not representative for TCR:pMHC complex structures.

Further, as Dunbar’s study focuses on the comparison of TCRs with antibodies, it is based on an adaption of the ABangle methodology to TCRs, which was originally developed for antibodies [24]. Although our method and ABangle have in common that they use the conserved positions in the IG-like domain for structural alignment, the ABangle method describes the rotation and translation by five angles, a distance, and a precomputed axis. A benefit of this method is the ability to inspect each component of the transformation separately and therefore it allowed identifying the main difference between the antibodies and the TCRs angular space, which lies in the HC2 (twist) angle. In contrast, the major goal of our analysis is to analyze possible orientations the two TCR variable domains can adopt depending on their type and state, functional mutations and the bound ligand. For this purpose we introduced a specialized robust method for the applied cluster analysis, which differs considerably from the method of Dunbar *et al*.: It reduces the variable domains to cuboids, to allow easy visualization of the transformational differences between two TCRs. Further, we describe the rotation of these cuboids by Euler angles, from which an Euclidean distance can be calculated, which is needed to obtain a robust clustering, as the commonly used RMSD-based measure was found to be too insensitive to capture the partially rather small angular differences between two TCRs. The Euler angle based measure showed a more robust performance and is, in addition, independent of protein translation. In contrary, the study of Dunbar *et al*. is based on the RMSD of the relative domain orientations, which is accurate enough to clearly distinguish between the two molecule classes (TCR and AB).

Finally, instead of several independent components we use one center of rotation (CoR) to describe the angular differences, which is a necessary prerequisite for the cuboid-based clustering and provides an intuitive measure for presenting and discussing our results. In addition, we use bootstrapping to confirm the significance of our clustering results [58].

As the focus of the study of Dunbar is on the comparison of the Vα/Vβ interdomain angles of TCRs and with the V_H_/V_L_ angles of antibodies, the study leads to the important result that TCRs and antibodies differ significantly in their interdomain angles. However, it also demonstrates that TCR-like antibodies, which were specially designed for pMHC binding, can adopt TCR-like geometries. Thus the study provides an important contribution to a better, detailed understanding of the structural features and characteristics of immunoglobulin-like folds and should therefore be very helpful for the rational of protein-based pharmaceuticals.

In agreement with the majority of the previous, predominantly experimental studies on small sets of TCRs, our comprehensive cluster analysis of the bound structures of the TCRs showed that TCRs of the same kind normally occupy the same structural cluster. Only one exception was observed, in this case two different clusters were found for the wt and mutant form (T7) of the same TCR (2C) and a specific combination of mutations in the framework and the CDR3 loop led to a shift of one mutant structure (T7-m67) into the cluster of the corresponding wt structures. For all other TCR types only one cluster was observed for both wt and mutated MHC-bound structures. These observations indicate that the differences in the Vα and Vβ interdomain angles of the bound TCR structures are predominantly determined by the preformed chain combination and subtype dependent interdomain angles of the unbound TCR structures and that neither the type of bound peptide nor the presenting MHC molecule lead to a significant angle shift. This geometry can be altered within the range of the subtype structures of the same TCR by mutations in the CDR loops as observed for the 2C m67 TCR. However, the analysis of the 1G4 structures showed that most changes in the CDR sequences do not have a significant effect on the interdomain angles. The same holds true for the binding of different MHC-peptide complexes to the same TCR, *e*.*g*. as shown for the A6 TCRs. This is in agreement with the previous studies of these TCRs, which observed only small differences in the Vα/Vβ interdomain angles for the different variants and bound pMHC complexes studies of these TCRs [14–16].

Comparing bound and unbound structures of the same TCR, a strong shift in the interdomain angles was observed in most cases upon binding of the TCR to a pMHC complex, as the bound and unbound structures of the same TCR were observed in different structural clusters. Further analysis showed that the differences in the interdomain Euler angles between the bound and unbound structures of the same TCR were often significantly higher than the variation of these angles within the bound or unbound structure set.

As basis for the observed differences in the association angles a so-called Center of Rotation (CoR) could be identified. This CoR is situated in the vicinity of two to four conserved residues (mainly Q), which interact via hydrogen bonding or charged interactions thus stabilizing the rotation center. Sequence analysis of these conserved residues showed that in contrary to antibodies in TCRs different amino acids can occupy these positions. However, all observed side chain types share the capability to form directed interactions such as hydrogen bonds. Due to the limited amount of TCR structures featuring other residues than Q at these positions, analysis of a correlation between the occurrence of specific residues at these position and the observed interdomain angles was not possible. The observation of a CoR is in agreement with previous studies of individual TCR types, as *e*.*g*. Ishizuka *et al*. [20] observed that for the JM22 TCR a binding hotspot of Vα/Vβ could be a center of motion or rocking. In this study, all JM22 structures were superimposed to the Vβ domains and the hinge was located at the salt bridge Q38α-Q39β [20]. In addition, already in the first described Vα/Vβ complex structure (2C) the (i) conserved Q-Q interaction between Vα and Vβ was observed at the Vα/Vβ binding interface, as well as water mediated hydrogen bonds between conserved residues of both domains and a symmetric hydrophobic core consisting of further conserved residues [26].

These individual results are considerably substantiated by our broad analysis. Throughout our dataset only minor variations were observed in the position of the CoR, which is highly conserved. In addition, nearly no shifting motions were observed, which seem to play only a minor role in the adjustment of the variable domains compared to the angular displacement.

Next to the conserved hydrogen bonds around the CoR, the contact area between the Vα and the Vβ domain is dominated by hydrophobic residues and is shaped similar to a saddle joint. This shape allows a certain rotation and translation of the Vβ domain sliding on the Vα-interface. As found by our grid analysis, the center of rotation is located at this area, but is slightly flexible. In contrast to the constant domains, the variable domains are not bound by a rigid disulfide bridge, but are kept together more loosely at the center of rotation by conserved Q-Q H-bond interactions. Our sequence analysis showed, that the Q-Q interaction is highly conserved, but in minor cases Q can be exchanged by other H-bond donors or acceptors or charged residues. Amino acids, which neither form H-bonds nor salt-bridges occur very seldom. In the latter case, the CoR possibly is shifted to other less conserved residues in this area, such as Y. Thus, the conserved residues at the CoR area keep the CoR at a defined position, but nevertheless the nature of the interactions permits flexibility that leads to the different orientations of the variable domains.

These observations are consistent with most other studies, discussing this topic, as a twofold hydrogen bond interaction between the Q-residues of the Vα and the Vβ domain was already reported for the first resolved TCR structure (1tcr, 2C) [26] and the involved residues are highly conserved for TCRs as well as for V_L_/V_H_ domains of antibodies [59]. Similarly, for antibodies it was proposed that in contrast to the constant domains the absence of a disulfide bond between the two variable domains is evolutionary preferred to allow for their rearrangement [38]. However, there exists one publication in which it was claimed that for A6 TCRs neither hydrogen bonds nor salt bridges can be observed between Vα/Vβ [13] and the authors propose that the diversity in Vα/Vβ rearrangement might be a result of the slippery hydrophobic interactions between the two variable domains. This is not only in contrast to the above discussed results from literature, but also our data, since we also observed the above described Q-Q interactions for all A6 TCRs (*e*.*g*. distances between the opposing atoms in structure 2gj6: D:Q37:NE2-E:Q37:OE1 = 3.06 Å, E:Q37:NE2-D:Q37:OE1 = 3.14 Å). Therefore, these electrostatic interactions, which are a magnitude weaker than a covalent disulfide bond, are highly conserved and most likely function as a flexible constraint, which keeps the two variable domains of the TCR in a preferred position, but at the same time allows for the necessary flexibility for their rearrangement upon binding to a specific pMHC complex.

Our analysis shows that TCRs of the same type bound to different ligands are normally found in the same clusters, whereas a significant change in the association angle can be observed upon binding of the TCR to the pMHC complex. Thus the question arises about the consequences of this behavior for the signal transduction cascade. According to our results two statements can be made. First, there seems to be a locking step upon pMHC assembly, during which the TCR is locked into a TCR clonotype specific geometry. Second, as the differences in the Vα/Vβ interdomain angles between the same TCRs bound to different binding partners, are rather small, the locking motion can be expected to be important during the signal transduction, whereas the differences in the absolute association angles are either not that significant or, assuming a signal is induced by the domain adjustment, only minor changes might be necessary. This agrees with most previous observations [22, 64], which show *e*.*g*. for the A6 TCR that peptide ligands with different affinities induce only minor changes in the relative positions of the variable domains [14–16]. In our analysis all A6 TCRs feature a very similar orientation of the Vβ domains and the structures all associate in the same cluster.

Due to its comprehensiveness our analysis puts these individual results on a common basis and provides thus a general picture of how pMHC binding influences TCR structure and function. Since many peptides with varying immunogenicity presented to the same TCR type only induce *minor* angular differences (see results), we conclude that signaling does not directly depend on a *major* change in the Vα/Vβ interdomain angle. In contrary, according to our analysis already minor changes of the Vα/Vβ interdomain geometry might have a significant influence on the triggering of the signaling cascade. These observations agree with the computational results of Knapp *et al*. who performed large scale MD simulations of 172 peptides of known immunogenicity presented to the LC13 TCR [56]. In that study, several features between a set of more immunogenic and less immunogenic peptides were compared, such as the Vα/Vβ geometries and the orientation of the TCR towards the pMHC, the solvent accessible surface area, the binding affinities, hydrogen bond footprints, and structural root mean square fluctuations (RMSF). The study confirms our results as the examined LC 13 TCR it was observed to adopt only a slightly more “open” binding site when recognizing more immunogenic peptides, which is consistent with the minor changes we see upon the binding of different ligands.

However, when discussing the topic of signal transduction, it needs to be stated that in our study we did not investigate positional changes of the constant domains. Such changes were observed in single studies and were postulated to have an influence on the minor changes in CD3 binding or activation [17]. On the other hand investigations of the relationships of constants domains of bound and unbound A6 TCRs showed no alteration [15]. However, possibly the conformational adjustment of the A6 constant domains might be very small. Different conformations of the Cα A-B loop dependent on the antigenic ligand were described and it was speculated that this alteration might induce the conformational changes of the CD3 molecule [69]. Possibly, the antigenic ligand induces first an adjustment of the variable domains, leading to a change of the relative positions of the constant domains and finally to the observed conformational change of the Cα A-B loop. This effect could either be achieved mechanically or by an alteration of the surrounding forces. For the JM22 TCR it was observed experimentally that the temperature factors of the constant TCR domains increase upon ligand binding [20]. This observation supports the idea of the Cα A-B loop becoming more flexible after other parts of the TCRs loose flexibility, as observed in this study through the locking motion upon pMHC binding. Regarding the structural analysis of the 2C TCR, this locking motion seems to be caused by interplay between the Vα/Vβ association angles and the bound-conformation of the MHC-binding CDR loops. MD simulations similar to the study of Knapp *et al*. [56] could be used to study these effects.

The structures of TCRs are generally similar to Fab-fragments of antibodies [14,26], whereby the AB V_L_/V_H_ correspond to the TCR Vα/Vβ domains. In early and recent studies of AB structures a rearrangement of the V_L_/V_H_ upon ligand binding was considered [25,27–39]. Computer-aided methods including MD simulations were carried out to investigate the changes of the elbow angle between the variable and the constant domains of antibodies [70–72]. TCRs feature a lower diversity in the variable loops 1 and 2 but a higher diversity in the CDR3 loop compared to ABs, resulting in a smaller diversity in the overall shape of the TCRs [14]. However, it was shown that ABs V_L_/V_H_ association angles are generally incompatible to the angular space of TCRs binding to MHC molecules [25]. It remains interesting to apply our method on ABs investigating whether this molecule class also shares a CoR and if ABs of the same type adapt to similar association angles.

### Conclusion

Since flexibility of the TCR Vα/Vβ interdomain association was considered for the first time in the end of the 90s of the last century [12], it took one decade to examine this phenomenon comparing the angular space of different TCR types due to the initial difficulties of obtaining experimental structures [22]. Now, immunologist can benefit from two independent new studies of the TCR interdomain association geometry by Dunbar *et al*. [25] and our present one. Both papers complement by focusing on different topics and methodologies. Whereas Dunbar *et al*. focus on the comparison between TCRs and antibodies, in this study we performed a systematic, exhaustive analysis of the Vα/Vβ interdomain angle for a representative set of experimental TCR structures. Our results are in agreement with the majority of previous experimental studies on small sets of TCRs. However, due to the comprehensiveness of our analysis we were able to put these individual observations on a broader, more general basis. This allowed us to deduce general features describing the relationship between TCR interdomain angle variations and pMHC binding and signaling.

First, our data clearly shows that the Vα/Vβ interdomain angle of pMHC-bound TCR structures can vary considerably, but is in most cases well conserved within the same TCR clonotype and its variants, independent of the ligand (pMHC) bound and individual mutations within the TCR. Nevertheless, there are individual exceptions like the 2C TCR, which show larger variations in their angle repertoire. Analysis of the 2C TCR structures revealed correlations between the Vα/Vβ interdomain angle, specific framework mutations, and conformational changes in the MHC-binding Vα-CDR loops due to Vα-CDR3 mutations. This is in accordance with previous experimental studies on individual TCRs, indicating that the Vα/Vβ interdomain angle is mainly influenced by the bound MHC allele and not the peptide. Unfortunately, due to the currently still sparse structural data available, no generalizable conclusions can be drawn about the dynamic mechanisms behind such Vα/Vβ angle switches.

Second, through a systematic analysis of the structural basis for the observed angular deviations we could identify a central point of rotation (CoR) common to all TCR structures independent of their state (bound or unbound) and type, which is stabilized by electrostatic and hydrogen bonding interactions. As in all previous studies the Vα/Vβ interdomain angle was described by at least three geometric quantities, the identification of one CoR, which allows a simple yet intuitive description of this functionally important, variable angle, sheds new light on the structural features and also the functional dynamics of TCRs and will also be important for the improvement of existing and for future TCR modeling approaches.

Third, analyzing bound versus unbound TCR structures, we observed that the angle variations between bound and unbound structures are more significant than between TCR structures bound to different MHC-peptide complexes or even mutated TCR structures with different specificities. This suggests that binding of the TCR to the pMHC complex is accompanied by a dynamic lock mechanism during which the two TCR variable domains are driven into a TCR-specific binding geometry leading to a stabilization of the TCR variable domain upon pMHC binding. In a previous study it was found that with a rigidification of the variable region the constant region becomes more flexible [20]. Furthermore, the influence of constant domain shifts was considered to be involved in CD3 activation [17] as well as a conformational change in the A-B loop of the Cα domain [69]. Supported by these observations we propose that locking of the variable domains upon ligand binding might enhance the motions of the constant domains in this oscillating system. The change of motion of the constant domains could then induce the conformational changes, which lead to CD3 activation and thus initiate T-cell signaling.

Based on these results the TCR/pMHC binding mechanism can be envisioned as a two-state process: First, preformation of the general α/β domain geometry in the free state and second, locking of this angle in a specific geometry upon association with the MHC-peptide complex. The last step might be an important feature during signal transduction upon binding.

## Methods

### Data set

A set of 85 X-ray crystal structures was acquired from the Protein Data Bank (PDB) [73]. The used structures contain bound and unbound TCRs from *H*. *sapiens* and *Mus musculus*. Receptors of invariant natural killer cells (iNKT) were not considered in our analysis. Although the iNKT receptors share sequential and structural similarity with other αβ TCRs, these special TCRs do not recognize pMHC ligands, but detect lipids presented by the MHC like CD1d molecule [74,75]. Thus iNKT:CD1d complexes must be treated separately. For the analysis each biological unit (BU) was treated as a separate structure, leading to a total amount of 136 different TCR complexes. For some analysis steps we computed averages over all BUs of the same crystal. This set is further referred to as *S*. The used structures are listed together with the names used in the literature and their bound state in Table 1. For some receptor types (*e*.*g*. A6, 1G4 etc.) several entries in the PDB are available. Furthermore, in some cases the names used in literature are ambiguous, since some of these structures bear mutations. Therefore, we compared the sequences of all structures against the wild type (wt), and we extended the TCR type names to indicate deviations. The sequence differences are presented in S1 Table and S2 Table in the supplementary material. This table also contains information about the appearing gene segments, differences in the CDR3 loops, and the bound ligands including the peptide sequence.

### Cuboid-based superpositioning approach

In a first preparatory step, all TCR structures were reduced to their variable binding domains (V). The constant domains of the TCRs were not considered for two reasons. First, in some of the TCR structures data are only available for the variable domains but not for the constant domains. Second, the constant and the variable domains are connected by a flexible loop. Superimpositioning the complete TCR chains onto their variable domains showed a visible displacement of the constant domains. Thus, including the constant domain into our superpositioning template would influence the structural alignment of the Vα domains.

### Cuboid construction and placement

Then we defined unified cuboids for each V domain of the different TCR chains. The cuboid templates (CTα and CTβ; Fig 1) comprise Vα or Vβ domain and a reduced set of the 2bnu Vα or Vβ domain framework residues. The reduced set was defined to allow for a robust superpositioning of the experimental structures during our future modeling procedure and during the geometrical measurements. For the superpositioning the tool DaliLite [76,77] was used together with the defined subset of V-framework residues as templates (Fig 1*C*). The Dali algorithm uses Cα-Cα distance matrices and does not depend on sequence information. Due to the high homology of the TCR framework regions, a solely structure based superpositioning method is indispensable for our needs.

To define the subset of residues contained in the superpositioning template, in a preparatory step the structures of each variable domain were superimposed separately. For this purpose all loops and turns were removed and the template residues were determined iteratively from the remaining residues, such that the set of mapped residues used as superpositioning anchors in DaliLite converged and the variance of the backbone root mean square deviation (RMSD) over all superposed structures was low. After the subsets were identified, the following procedure was used to superpose the combined variable domains and to place the cuboids. First, the Vα:Vβ-complexes were superimposed based on their Vα domains using the above defined α-subset and the tool DaliLite. All structures were superimposed to the high resolution (1.4 Å) structure with the PDB ID 2bnu. This step leads to a set of TCR-structures, which is further referred to as Sα and a corresponding set of cuboid templates (CTα), containing cuboids, which were placed around the Vα domains based on the positions of the α-subset residues. Second, the same procedure was used to place cuboid templates (CT, Fig 1*C*) additionally around each Vβ domain contained in the set Sα according to the relative position of the β chains towards their paired, superposed Vα domains resulting in a set of cuboid templates around the Vβ domains (CTβ). This step results in the set C consisting of the Vβ domain cuboids (CTβ) and the corresponding Vβ domain structures.

### Comparison of the relative Vα/Vβ domain geometries

We computed the Euler angles for each cuboid-based geometry with respect to a reference coordinate system. The calculation was implemented using the GNU generic math template library; all angles were computed in xyz-order. The reference coordinate system was chosen to be the coordinate system of the 2bnu structure (Fig 1*C*). Since all structures of the set S were superimposed to conserved framework residues of the reference structure 2bnu, the rotation of the Vβ domain is computed relative to the orientation of the Vβ of 2bnu. The similarity between two geometries we defined as the Euclidean distance of the Euler angles [78]:

$$d_{E}\left(i,j\right)=\sqrt{{\left(\Phi_{i}-\Phi_{j}\right)}^{2}+{\left(\Psi_{i}-\Psi_{j}\right)}^{2}+{\left(\Theta_{i}-\Theta_{j}\right)}^{2}}$$ (1)

The distance matrix D was clustered hierarchically, using Ward’s minimum variance method [57,79]. In the case of structures containing multiple BUs each Euler angle component Φ, Ψ, and θ was averaged, leading to an artificial unified geometry. These unified averaged geometries cluster together with other TCRs of the same type, describing a general geometrical state of a TCR. In contrast, the differing geometries found within one structure indicate, that TCRs may adopt an ensemble of different geometries. The significance of the found clusters was confirmed using a bootstrap analysis [58]. The number of bootstrap replica was set to 10^6^.

### Center of rotation and translation

The common center of rotation (CoR) was computed for all structures (BUs were treated independently), and subsequently the residues situated in the CoR-areas were analyzed. The conservation of these CoR-residues was confirmed by multiple sequence alignment of the TCR.

The CoR was determined for each Vβ domain of the set *C*. For this purpose, grids were fitted into the cuboids (as illustrated in Fig 1*D*) and the grid points were indexed in the same manner for each cuboid. For all grid points of the same index *i*, pairwise distances were computed. The variance

$$\mathrm{var}(g_{i})=\frac{1}{n^{2}-1}\sum_{k=1}^{n}\sum_{l=1}^{n}\left(\delta_{S}\left(g_{i,k},g_{i,l}\right)-\frac{\sum_{r=1}^{n}\sum_{s=1}^{n}\delta_{S}\left(g_{i,r},g_{i,s}\right)}{n^{2}}\right)$$ (2)

was determined; *δ*_*S*_(*g*_*i*,*x*_,*g*_*i*,*y*_) is the Euclidean spatial distance between a grid point with the index *i* of the structure *x* and the equivalent grid point of structure *y*; *n* denotes the number of observed structures. We define the CoR grid index point as *I*_*min*_ = argmin(min{var(*g*_*i*_)|1 ≤ *i* ≤ *n*}). The corresponding coordinate for *I*_min_ was computed according to the reference coordinate system of the reference structure 2bnu. The computations were performed on the whole set *C* and on subsets of this set. The first subset *C* ^*b*^ contained only MHC bound TCRs, whereas the second subset *C* ^*u*^ contained unbound TCRs. Furthermore, we investigated the structural environment of the location of the corresponding *I*_min_ coordinates. The grids were implemented in a cubic shape with an amount of 33,076,161 grid points and a minimum distance between each point of 0.1 Å, resulting in a grid size of 32 Å in each dimension. In contrast to the intersect method for the CoR calculation, the grid method allows highlighting invariant areas and is more robust against deviations caused by geometrical translation. Sequence similarity and conservation of amino acids located at the CoR was explored by creating multiple sequence alignments (MSAs) of all known human and murine (functional) TCR variable segment sequences. For this purpose, the tool MAFFT [80] (linsi: localpair, maxiter 1000, Blosum62 [81]) was applied on sequences obtained from the IMGT GENE-DB [61].

## Supporting Information

S1 Text Analysis of specific TCR:pMHC structures: Detailed discussion for the individual TCR types. (PDF)

S1 Table Properties of available TCR structures. For each X-ray structure TCR names used in the literature are listed and the relevant TCR chains within one biological unit are indicated. Loci and alleles of the TRAV, TRBV, TRAJ, and TRBJ were assigned using the IMGT Gene/DB [61] and the sequences of the CDR loops are listed for comparison of the different subtypes (Mutations within the framework region are summarized separately in S2 Table). For the bound TCRs the loci/allele of the MHC (-like) molecule and it’s ligand is provided; mutations are indicated.
(PDF)

S2 Table Mutations within the framework region of the TCR structures. The sequences of the TCR structures were compared to the corresponding wild type (WT). Differences to the alleles provided in the IMGT Gene-DB [61] are provided as mutation pairs in brackets. Naming of the sheets and loops, and the residue indices follow the IMGT unique numbering [62].
(PDF)

S3 Table Multiple sequence alignments at the COR. (PDF)

S4 Table Epitopes of the bound TCR structures. (PDF)

S5 Table References to the TCR structures used for the analysis. (PDF)

S1 Fig Bootstrapping dendrogram of the clustering of the MHC bound TCRs. Pairwise Euler-angle distances (EAD) were determined for all MHC-bound TCR structures and the free TCR structures according to Formula 1. Structures containing more than one biological unit were merged to one unique geometry. The distance matrix was hierarchically clustered using the Ward update formula. For each subtree of the dendrogram, the au (approximately biased) and the bp (bootstrapping probability) according to the bootstrapping method [58] are provided. We identified six significant clusters of an au-value greater than 95%. The clusters are marked by colored boxes.
(PDF)

S2 Fig Bootstrapping dendrogram of the clustering of the free TCRs together with the MHC bound TCRs. For details see S1 Fig. The bootstrapping dendrogram was computed for the bound and free TCRs. The clusters of the unbound case are marked by colored boxes for comparison. Significant clusters are only found for smaller subtrees.
(PDF)

S3 Fig Geometry clusters of bound and unbound TCRs. Pairwise Euler-angle distances (EAD) were determined for all MHC-bound TCR structures and the free TCR structures according to Formula 2 (see Methods). Structures containing more than one biological unit were merged to one unique geometry. The distance matrix was hierarchically clustered using the Ward update formula. We identified six significant clusters, using a bootstrapping approach [58]. Notably, in most of the cases, TCRs of the same type occur in the same cluster. Upper panel: Clustering dendrogram with bootstrapping results (au = approximately unbiased, bp = bootstrapping probability) Left panel: TCR types occurring within a cluster. Right/lower panel: Structure PDB identifiers and corresponding TCR names. MHC unbound TCR structures are indicated by bold-italics fonts. Central panel: Pairwise Euler-angle distances (EAD). The color key is provided in the bottom of the figure.
(PDF)
