# Supplementary material for: Quantitative Analysis of the Association Angle between T-cell Receptor Vα/Vβ Domains Reveals Important Features for Epitope Recognition
Source: PLoS Comput Biol. 2015 Jul 17;11(7):e1004244. doi: 10.1371/journal.pcbi.1004244 (PMC4505886; doi:10.1371/journal.pcbi.1004244)
Supplement: S1 Text — (PDF) [file pcbi.1004244.s001.pdf]

S1 Text. Analysis of specific TCR:pMHC structures: Detailed discussion for the individual TCR types

In addition to the comparative cluster analysis we performed a more detailed functional and structural analysis of the clustering results. For several of the analysed TCR types there exist different structures in which the TCR is either bound to different MHC alleles and/or different peptides or different variants of the same TCR were crystallized. Analyzing the clustering behavior of these different structures of the same TCR types and their variants we observed that all except one (2C) are located in the same clusters.

Thus, we investigated the 2C TCR geometries in more detail. A closer look at the alloreactive murine 2C receptor shows that it can be observed in two different clusters (Figure 2: orange cluster (4) and cyan cluster (6)), whereas for all other TCR types all structures are members of the same cluster. The repertoire of the resolved 2C TCR structures is quite large in comparison to the other TCRs and contains different mutants of this receptor as well as different ligands (1tcr, 1g6r, 1mwa, 2ckb 2icw, 2oi9, 3e3q, 2e7l, 3e2h) [1-7] In Table 3 all pairwise Euler angle distances are provided together with the receptor's subtype and its ligand. We observe clustering in two significantly different geometries. The 2C structure repertoire contains wild type (2C wt) TCRs (1tcr, 1g6r, 1mwa, 2ckb), as well as four different variants of the 2C T7 TCR (2oi9, 3e3q, 2e7l, 3e2h). Next to several MHC-bound structures the 2C wt TCR is also available in its free state (1tcr).

In this context we would like to mention that the 2C T7 TCR crystal structure 2icw [7], in which the receptor is bound to a superantigen in association with a human MHC class II molecule, was not considered in this analysis, because in this structure direct TCR:MHC interaction is prevented by the superantigen which due to its size bridges all interactions. Therefore, this structure has a special binding mode, which is different from all other structures in our analysis and was thus discarded. The studied 2C T7 TCR variants (m13, m16, and m67) share the same mutations in the αC', αE, βB, and βC' strands (Table S2) with respect to the 2C wt TCR sequence as the original T7 variant, but additionally differ in their sequences of the Vα-CDR3 loop (Table S1), whereas no such differences exist in the original T7 variant. The MHC-bound 2C wt structures are crystallized together with different murine MHC class I molecules (H2-K^b (2ckb) or it's mutant form H2-K^{bm3} (1mwa)) and different bound peptides (EQYKFYSV (1mwa, 2ckb) or SIYRYYGL (1g6r)). The MHC-bound T7 (2oi9, 3e3q, 2e7l, 3e2h) structures are all associated with the same murine MHC class I molecule H2-L^d and the peptide QLSPFPFDL. Regarding Figure 2 it can be observed that all 2C wt TCR (2ckb, 1mwa, 1g6r) structures are members of cluster 6, whereas the T7 structures of the variants m6, m13, and T7-wt (3e3q, 2e7l, 2oi9) associate in cluster 4. However, the T7 structure of the m67 (3e2h) variant is member of cluster 6 along with the 2C wt TCRs, *i.e.* the m67 variant structure has the same Vα/Vβ association angle as the 2C wt TCR and not as the T7 TCRs. To elucidate the structural reason behind the structural differences within the 2C TCR structures a thorough structural and sequence analysis of all 2C structures was performed:

First, to investigate the potential influence of the different pMHC complexes on the structural differences in the TCRs, we analyzed the pMHC complexes to which the 2C wt and T7 structures are bound (Table 3). It can be observed that all 2C wt TCRs are bound to different MHC alleles as well as different peptide molecules, however, these do not influence the TCR geometry. In contrast, all T7 structures are bound to

the same pMHC complex and feature the same Vα/Vβ angle, except the m67 variant, which adopts the 2C wt geometry although the 2c wt TCRs are bound to a different pMHC complex. Thus the data set does not allow any judgment if the observed differences are caused by different pMHC alleles or rather by the differences within the TCR variants.
Second, analyzing the structures in more detail it can first be observed that the CDR loops of the Vβ domains adopt similar conformations for all 2C and T7 structures and show the same interaction geometry with the peptide and the MHC molecule. This is in contrast to the CDR1 and CDR3 loop conformations of the Vα domains, which differ between the 2C and T7 structures (Figure 4B), leading to different pMHC binding geometries and thus differences in the overall Vα/Vβ angle. Considering that the 2C wt and T7 structures only differ sequence-wise in the framework region, mutations in this region seem to have a direct influence on the TCRs CDR conformations.
Third, in the cases of the T7 variants, all variants are only mutated in their CDR3 loops and for the m6 and m13 variants, the mutations in their Vα-CDR3 loop only alter the conformation of this loop, but leave the Vα-CDR1 loop in its original T7 conformation. In contrary in the m67 variant, the conformational changes in its Vα-CDR3 loop lead to structural changes in its Vα-CDR1 loop, which adopts the conformation of the 2C wt structure (Figure 4B). As the m67 variant is the only T7 variant, which adopts the 2C wt Vα/Vβ angle, the CDR1 conformation seems to play a crucial role for the Vα/Vβ association angle, whereas the CDR3 conformation influences the angle only indirectly. Therefore, two effects could be observed within the available 2C TCR structural ensemble: Differences in the TCR framework sequences can lead to different Vα/Vβ association angles which can cause different CDR loop conformations and altered pMHC binding. On the other hand, differences in the sequences of the CDR loops, which lead to changes in the MHC binding CDRs (1 and 2) can back-lead to differences in the Vα/Vβ association angles.
A second TCR type, 1G4, for which various structures are available, contains several subtypes (Table S1 and S2), which differ in the αCDR1, αCDR3, βCDR1, and the βCDR3 loops. In contrast to the 2C variants above, for the 1G4 TCR variants cluster affiliations do not differ. Thus, in this case, all TCR structures (2bnu, 2bnq, 2bnr, 2f54, 2pyf, 2pye, 2f53, 2p5w, 2p5e), which are all bound to HLA-A*0201, are accumulated in cluster 1. In all structures the receptors are bound to the peptide SLMWITQC, except in the wild type (wt) structure 2bnq, which contains the peptide SLMWITQV.
In the case of the A6 TCR all nine structures (2gj6, 1qsf, 1qse, 3d3v, 3d39, 1qrn, 1ao7, 3h9s, 3pwp) can be found in the same cluster (cluster 2). The averaged angular distance between the nine structures is 2.1º (var=0.8º). All these structures share exactly the same TCR bound to HLA-A*0201, but differ in the bound peptide. The two structures 3d39 and 3d3v both contain (double)fluorinated derivatives of the peptide mutant LLFGFPVYV to achieve an increased affinity ($y5f^{4F}$ or $y5f^{3,4FF}$) [8]. Furthermore, in the structure 2gj6 [9] the peptide LLFGKFVYV, where the K-residue is linked to 4-(3-indolyl)-butric acid, is presented.
The three BM3.3 bound structures differ in average by 3.1º and can be found in cluster 5. The structures mainly differ by the presented peptides (SQYYYNSL, INFDFNTI, and RGYVYQGL) presented by the MHC molecules $H2\text{-}K1^{b}$ or $H2\text{-}K1^{bm8}$. Similar to the case of the A6 TCRs; the different peptides do not significantly alter the TCR geometry.

For the JM22 TCR four different structures (2vlj, 2vlk, 2vlr, and 1oga) with pMHC ligands are available. All of them share the same ligand, which is the peptide GILGFVFTL presented by the MHC molecule HLA-A*0201. The sequences of the TCRs of the four structures do not differ, except for 2vlr, which contains the mutation S99A in the αCDR3-loop. The average angular distance between all 4 structures is low (1.5º) and as expected they associate all in the same cluster (cluster 3).
A set of AHIII12.2 (wt) TCRs (2jcc, 2uwe, and 1lp9), can be found in cluster 6. To the three TCRs the peptide ALWGFFPVL is presented by the MHC molecule HLA-A*0201. In two cases a single amino acid is mutated within the binding pockets of the MHC molecules (2jcc: W176V, 2uwe: T163A) [10]. However, the differences in the MHC binding pockets obviously do not significantly alter the TCR geometry (average angular distance: 1.3º)
Three different structures of the TK3 TCR are available, the wt structure (3mv7), the mutant Q55H (3mv8), and the mutant Q55A (3mv9), all bound to a HLA-B*3501:HPVGEADYFEY complex. The mutations of the two variants are located in the αC'-sheet of the framework region. This allelic polymorphism (TRBV9*01 vs. TRBV9*02) influences the charge complementarity at the binding interface to the pMHC ligand [11]. Although these framework mutations alter the activity of the TCR, the exchange of Q55 to H or A does not influence the TCR interdomain geometry: all TK3 structures associate in cluster 6 (average angular distance: 0.7º).
The two different TCRs 2B4 (3qib) and 226 (3qiu, 3qiw) can both be found in cluster 4. Both TCRs bind to the murine MHC class II molecule H2-Ea^{k}:H2-Eb^{k} either presenting the peptide ADLIAYLKQATKG (3qib, 3qiu) or ADLIAYLEQATKG (3qiw). The 2B4 TCR facilitates the alleles TRAV4D-4*02, TRAJ56*01, TRBV26*01, and TRBJ2-5*01, whereas the 226 TCR uses the alleles TRAV4D-4*01, TRAJ16*01, TRBV26*01, and TRBJ1-2*01 (Table S1). Thus, the different TCRs have a similar framework region in common and share the CDR1/2 loops of both chains. Even though, both receptors differ in their CDR3 loops and the J-segments and an averaged angular distance of only 1.5º can be measured.
More TCRs of the same type in the same cluster are listed in Table S4, but are not further discussed, since for each of them only two representatives are available (E8 (cl. 1), SB27 (cl. 3), OB1A12 (cl. 3), and HA1.7 (cl. 5)).

SI References:

1. Colf LA, Bankovich AJ, Hanick NA, Bowerman NA, Jones LL, et al. (2007) How a single T cell receptor recognizes both self and foreign MHC. Cell 129: 135-146.
2. Degano M, Garcia KC, Apostolopoulos V, Rudolph MG, Teyton L, et al. (2000) A functional hot spot for antigen recognition in a superagonist TCR/MHC complex. Immunity 12: 251-261.
3. Garcia KC, Degano M, Pease LR, Huang M, Peterson PA, et al. (1998) Structural basis of plasticity in T cell receptor recognition of a self peptide-MHC antigen. Science 279: 1166-1172.
4. Garcia KC, Degano M, Stanfield RL, Brunmark A, Jackson MR, et al. (1996) An alphabeta T cell receptor structure at 2.5 A and its orientation in the TCR-MHC complex. Science 274: 209-219.
5. Jones LL, Colf LA, Stone JD, Garcia KC, Kranz DM (2008) Distinct CDR3 conformations in TCRs determine the level of cross-reactivity for diverse antigens, but not the docking orientation. J Immunol 181: 6255-6264.

6. Luz JG, Huang M, Garcia KC, Rudolph MG, Apostolopoulos V, et al. (2002) Structural comparison of allogeneic and syngeneic T cell receptor-peptide-major histocompatibility complex complexes: a buried alloreactive mutation subtly alters peptide presentation substantially increasing V(beta) Interactions. J Exp Med 195: 1175-1186.
7. Wang L, Zhao Y, Li Z, Guo Y, Jones LL, et al. (2007) Crystal structure of a complete ternary complex of TCR, superantigen and peptide-MHC. Nat Struct Mol Biol 14: 169-171.
8. Piepenbrink KH, Borbulevych OY, Sommese RF, Clemens J, Armstrong KM, et al. (2009) Fluorine substitutions in an antigenic peptide selectively modulate T-cell receptor binding in a minimally perturbing manner. Biochem J 423: 353-361.
9. Gagnon SJ, Borbulevych OY, Davis-Harrison RL, Turner RV, Damirjian M, et al. (2006) T cell receptor recognition via cooperative conformational plasticity. J Mol Biol 363: 228-243.
10. Miller PJ, Pazy Y, Conti B, Riddle D, Appella E, et al. (2007) Single MHC mutation eliminates enthalpy associated with T cell receptor binding. J Mol Biol 373: 315-327.
11. Gras S, Chen Z, Miles JJ, Liu YC, Bell MJ, et al. (2010) Allelic polymorphism in the T cell receptor and its impact on immune responses. J Exp Med 207: 1555-1567.
