# Supplementary material for: Quantitative Analysis of the Association Angle between T-cell Receptor Vα/Vβ Domains Reveals Important Features for Epitope Recognition
Source: PLoS Comput Biol. 2015 Jul 17;11(7):e1004244. doi: 10.1371/journal.pcbi.1004244 (PMC4505886; doi:10.1371/journal.pcbi.1004244)
Supplement: S1 Table — For each X-ray structure TCR names used in the literature are listed and the relevant TCR chains within one biological unit are indicated. Loci and alleles of the TRAV, TRBV, TRAJ, and TRBJ were assigned using the IMGT Gene/DB [61] and the sequences of the CDR loops are listed for comparison of the different subtypes (Mutations within the framework region are summarized separately in S2 Table). For the bound TCRs the loci/allele of the MHC (-like) molecule and it’s ligand is provided; mutations are indicated. (PDF) [file pcbi.1004244.s002.pdf]

S1 Table. Properties of available TCR structures. For each X-ray structure TCR names used in the literature are listed and the relevant TCR chains within one biological unit are indicated. Loci and alleles of the TRAV, TRBV, TRAJ, and TRBJ were assigned using the IMGT Gene/DB [1] and the sequences of the CDR loops are listed for comparison of the different subtypes (Mutations within the framework region are summarized separately in Table S2). For the bound TCRs the loci/allele of the MHC (-like) molecule and it's ligand is provided; mutations are indicated.

Name	Bound State[a]	PDB-ID	Chain Pairs	Spec.	Literature PMID	Alpha IMGT-Name V-Segment	Alpha IMGT-Name J-Segment	Alpha CDR Loops[b] 1	Alpha CDR Loops[b] 2	Alpha CDR Loops[b] 3	Beta IMGT-Name V-Segment	Beta IMGT-Name J-Segment	Beta CDR-Loops[b] 1	Beta CDR-Loops[b] 2	Beta CDR-Loops[b] 3	Ligand Pep/Lip[c]	Ligand MHC I/II alpha	Ligand MHC II beta
1G4	u	2BNU	AB	H	15837811	TRAV21*01	TRAJ6*01	DSA......IYN	IQSS...QRE	AVRPTSGGSYIPT	TRBV6-5*01	TRBJ2-2*01	MNH.......EY	SVG....AGI	ASSYVG.NTGELF			
1G4	1	2BNQ	DE	H	15837811	TRAV21*01	TRAJ6*01	DSA......IYN	IQSS...QRE	AVRPTSGGSYIPT	TRBV6-5*01	TRBJ2-2*01	MNH.......EY	SVG....AGI	ASSYVG.NTGELF	SLLMWITQV	HLA-A*0201	
1G4	1	2BNR	DE	H	15837811	TRAV21*01	TRAJ6*01	DSA......IYN	IQSS...QRE	AVRPTSGGSYIPT	TRBV6-5*01	TRBJ2-2*01	MNH.......EY	SVG....AGI	ASSYVG.NTGELF	SLLMWITQC	HLA-A*0201	
1G4 AV-wt	1	2F54	DE,KL	H	16600963	TRAV21*01	TRAJ6*01	DSA......IYN	IQSS...QRE	AVRPTSGGSYIPT	TRBV6-5*01	TRBJ2-2*01	MNH.......EY	SVG....AGI	ASSYVG.NTGELF	SLLMWITQC	HLA-A*0201	
1G4 c5c1	u	2PYF	AB	H	17644531	TRAV21*01	TRAJ6*01	DSA......IYN	IQSS...QRE	AVRPLLDGTYIPT	TRBV6-5*01	TRBJ2-2*01	MNH.......EY	SVG....AGT	ASSYLG.NTGELF			
1G4 c5c1	1	2PYE	DE	H	17644531	TRAV21*01	TRAJ6*01	DSA......IYN	IQSS...QRE	AVRPLLDGTYIPT	TRBV6-5*01	TRBJ2-2*01	MNH.......EY	SVG....AGT	ASSYLG.NTGELF	SLLMWITQC	HLA-A*0201	
1G4 c49c50	1	2F53	DE	H	16600963	TRAV21*01	TRAJ6*01	DSA......IYN	IPFW...QRE	AVRPTSGGSYIPT	TRBV6-5*01	TRBJ2-2*01	MNH.......EY	SVS....VGM	ASSYVG.NTGELF	SLLMWITQC	HLA-A*0201	
1G4 c58c62	1	2P5W	DE	H	17644531	TRAV21*01	TRAJ6*01	DSA......IYN	ITPW...QRE	AVRPLLDGTYIPT	TRBV6-5*01	TRBJ2-2*01	MNH.......EY	SVS....VGM	ASSYLG.NTGELF	SLLMWITQC	HLA-A*0201	
1G4 c58c61	1	2P5E	DE	H	17644531	TRAV21*01	TRAJ6*01	DSA......IYN	ITPW...QRE	AVRPLLDGTYIPT	TRBV6-5*01	TRBJ2-2*01	MNH.......EY	SVA....IQT	ASSYLG.NTGELF	SLLMWITQC	HLA-A*0201	
3A6	2	1ZGL	ST,MP,QR, UV	H	16079912	TRAV9-2*02	TRAJ12*01	ATG......YPS	ATKA...DDK	ALSGGD.SSYKLI	TRBV5-1*01	TRBJ1-1*01	SGH.......RS	YFN....ETQ	ASSLADRVNTEAF	VHFFKNIVTPRTP	HLA-DRA*0101	HLA-DRB5*0101
A6	1	2GJ6	DE	H	16962135	TRAV12-2*02	TRAJ24*02	DRG......SQS	IYS....NGD	AVTTDS..WGKLQ	TRBV6-5*01	TRBJ2-7*01	MNH.......EY	SVG....AGI	ASRPGLAGGRPEQY	LLFGKPVYV [d]	HLA-A*0201	
A6	1	1QSF	DE	H	10435578	TRAV12-2*02	TRAJ24*02	DRG......SQS	IYS....NGD	AVTTDS..WGKLQ	TRBV6-5*01	TRBJ2-7*01	MNH.......EY	SVG....AGI	ASRPGLAGGRPEQY	LLFGYPVAV	HLA-A*0201 [e]	
A6	1	1QSE	DE	H	10435578	TRAV12-2*02	TRAJ24*02	DRG......SQS	IYS....NGD	AVTTDS..WGKLQ	TRBV6-5*01	TRBJ2-7*01	MNH.......EY	SVG....AGI	ASRPGLAGGRPEQY	LLFGYPRYV	HLA-A*0201	
A6	1	3D3V	DE	H	19698083	TRAV12-2*02	TRAJ24*02	DRG......SQS	IYS....NGD	AVTTDS..WGKLQ	TRBV6-5*01	TRBJ2-7*01	MNH.......EY	SVG....AGI	ASRPGLAGGRPEQY	LLFGFPVYV [n]	HLA-A*0201	
A6	1	3D39	DE	H	19698083	TRAV12-2*02	TRAJ24*02	DRG......SQS	IYS....NGD	AVTTDS..WGKLQ	TRBV6-5*01	TRBJ2-7*01	MNH.......EY	SVG....AGI	ASRPGLAGGRPEQY	LLFGFPVYV [o]	HLA-A*0201	
A6	1	1QRN	DE	H	10435578	TRAV12-2*02	TRAJ24*02	DRG......SQS	IYS....NGD	AVTTDS..WGKLQ	TRBV6-5*01	TRBJ2-7*01	MNH.......EY	SVG....AGI	ASRPGLAGGRPEQY	LLFGYAVYV	HLA-A*0201	
A6	1	1AO7	DE	H	8906788	TRAV12-2*02	TRAJ24*02	DRG......SQS	IYS....NGD	AVTTDS..WGKLQ	TRBV6-5*01	TRBJ2-7*01	MNH.......EY	SVG....AGI	ASRPGLAGGRPEQY	LLFGYPVYV	HLA-A*0201	
A6	1	3H9S	DE	H	20064447	TRAV12-2*02	TRAJ24*02	DRG......SQS	IYS....NGD	AVTTDS..WGKLQ	TRBV6-5*01	TRBJ2-7*01	MNH.......EY	SVG....AGI	ASRPGLAGGRPEQY	MLWGYLQYV	HLA-A*0201	
A6	1	3PWP	DE	H	21282516	TRAV12-2*02	TRAJ24*02	DRG......SQS	IYS....NGD	AVTTDS..WGKLQ	TRBV6-5*01	TRBJ2-7*01	MNH.......EY	SVG....AGI	ASRPGLAGGRPEQY	LGYGFVNYI	HLA-A*0201	
AS01	1	3O4L	DE	H	21124993	TRAV5*01	TRAJ31*01	DSS......STY	IFSN...MDM	AEDNN....ARLM	TRBV20-1*01	TRBJ1-2*01	DFQ......ATT	SNEG...SKA	SARDGT..GNGYT	GLCTLVAML	HLA-A*0201	
B7	1	1BD2	DE	H	9586631	TRAV29*01	TRAJ54*01	NSM......FDY	ISSI...KDK	AAMEG...AQKLV	TRBV6-5*01	TRBJ2-7*01	MNH.......EY	SVG....AGI	ASSYPGGGFYEQY	LLFGYPVYV	HLA-A*0201	
cf34	1	3FFC	DE,KJ	H	19167249	TRAV14*01	TRAJ49*01	TSDP.....SYG	QGSY..DQGN	AMREDT..GNQFY	TRBV11-2*01	TRBJ2-3*01	SGH.......AT	FQN....NGV	ASSFTWTSGGATD	FLRGRAYGL	HLA-B*0801	
DM1	1	3DXA	DE,IJ,NO	H	19139173	TRAV26-1*02	TRAJ13*02	TISG.....NEY	GLK.....NN	IVWGG...YQKVT	TRBV7-9*01	TRBJ2-1*01	SEH.......NR	FQN....EAQ	ASRYRDDSYNEQF	EENLLDFVRF	HLA-B*4405	
DM1	u	3DX9	AB,CD	H	19139173	TRAV26-1*02	TRAJ13*02	TISG.....NEY	GLK.....NN	IVWGG...YQKVT	TRBV7-9*01	TRBJ2-1*01	SEH.......NR	FQN....EAQ	ASRYRDDSYNEQF			
E8	2	2IAN	DE,IJ,NO,ST	H	17334368	TRAV22*01	TRAJ54*01	DSV.......NN	IPS.....GT	AALIQG..AQKLV	TRBV6-6*01	TRBJ1-3*01	MNH.......NY	SVG....AGI	ASTYH....GTGY	GELIGTLNAAKVPAD	HLA-DRA*0101	HLA-DRB1*0101
E8	2	2IAM	CD	H	17334368	TRAV22*01	TRAJ54*01	DSV.......NN	IPS.....GT	AALIQG..AQKLV	TRBV6-6*01	TRBJ1-3*01	MNH.......NY	SVG....AGI	ASTYH....GTGY	GELIGILNAAKVPAD	HLA-DRA*0101	HLA-DRB1*0101
E8	u	2IAL	AB,CD	H	17334368	TRAV22*01	TRAJ54*01	DSV.......NN	IPS.....GT	AALIQG..AQKLV	TRBV6-6*01	TRBJ1-3*01	MNH.......NY	SVG....AGI	ASTYH....GTGY			
ELS4	1	2NX5	DE,IJ,NP,TU	H	17259989	TRAV1-2*01	TRAJ6*01	TSG......FNG	NVL....DGL	AVQASG.GSYIPT	TRBV10-3*01	TRBJ1-5*01	ENH.......RY	SYG....VKD	ATGTGD.SNQPQH	EPLPQGQLTAY	HLA-B*3501	
ELS4	u	2NW2	AB	H	17259989	TRAV1-2*01	TRAJ6*01	TSG......FNG	NVL....DGL	AVQASG.GSYIPT	TRBV10-3*01	TRBJ1-5*01	ENH.......RY	SYG....VKD	ATGTGD.SNQPQH			
HA1.7	2	1FYT	DE	H	11060013	TRAV8-4*05	TRAJ48*01	SSV......PPY	YTSA..ATLV	AVSESPFGNEKLT *	TRBV28*01	TRBJ1-2*01	MDH.......EN	SYD....VKM	ASSSTG.LPYGYT	PKYVKQNTLKLAT	HLA-DRA*0101	HLA-DRB1*0101
HA1.7	2	1J8H	DE	H	11877480	TRAV8-4*05	TRAJ48*01	SSV......PPY	YTSA..ATLV	AVSESPFGNEKLT *	TRBV28*01	TRBJ1-2*01	MDH.......EN	SYD....VKM	ASSSTG.LPYGYT	PKYVKQNTLKLAT	HLA-DRA*0101	HLA-DRB1*0401
Hy.1B1	2	3PL6	CD	H	21199956	TRAV13-1*02	TRAJ48*01	DSA......SNY	IRSN...VGE	AASSFG..NEKLT *	TRBV7-3*01	TRBJ2-3*01	SGH.......TA	FQG....TGA	ATSAL...GDTQY	NPVVHFFKNIVTPR (L)	HLA-DQA1*0102	HLA-DQB1*0501
JM22	1	2VLJ	DE	H	18275829	TRAV27*01	TRAJ42*01	SVF.......SS	VVTG...GEV	AGAGS...QGNLI	TRBV19*01	TRBJ2-7*01	LNH.......DA	SQI....VND	ASSSRS..SYEQY	GILGFVFTL	HLA-A*0201	
JM22	1	2VLK	DE	H	18275829	TRAV27*01	TRAJ42*01	SVF.......SS	VVTG...GEV	AGAGS...QGNLI	TRBV19*01	TRBJ2-7*01	LNH.......DA	SQI....VND	ASSSRS..SYEQY	GILGFVFTL	HLA-A*0201	
JM22	1	1OGA	DE	H	12796775	TRAV27*01	TRAJ42*01	SVF.......SS	VVTG...GEV	AGAGS...QGNLI	TRBV19*01	TRBJ2-7*01	LNH.......DA	SQI....VND	ASSSRS..SYEQY	GILGFVFTL	HLA-A*0201	
JM22	u	2VLM	DE	H	18275829	TRAV27*01	TRAJ42*01	SVF.......SS	VVTG...GEV	AGAGS...QGNLI	TRBV19*01	TRBJ2-7*01	LNH.......DA	SQI....VND	ASSSRS..SYEQY			
JM22	2,s	2XN9	AB	H	21081917	TRAV27*01	TRAJ42*01	SVF.......SS	VVTG...GEV	AGAGS...QGNLI	TRBV19*01	TRBJ2-7*01	LNH.......DA	SQI....VND	ASSSRS..SYEQY	PKYVKQNTLKLAT	HLA-DRA*0101	HLA-DRB1*0101
JM22	s	2XNA	AB	H	21081917	TRAV27*01	TRAJ42*01	SVF.......SS	VVTG...GEV	AGAGS...QGNLI	TRBV19*01	TRBJ2-7*01	LNH.......DA	SQI....VND	ASSSRS..SYEQY			
JM22 [S99βA]	1	2VLR	DE,IJ	H	18275829	TRAV27*01	TRAJ42*01	SVF.......SS	VVTG...GEV	AGAGS...QGNLI	TRBV19*01	TRBJ2-7*01	LNH.......DA	SQI....VND	ASSSRA..SYEQY	GILGFVFTL	HLA-A*0201	
KK50.4	1	2ESV	DE	H	16474394	TRAV26-1*01	TRAJ37*01	TISG.....NEY	GLK.....NN	IVVRSS.NTGKLI	TRBV14*01	TRBJ2-3*01	SGH.......DN	FVK....ESK	ASSQD...RDTQY	VMAPRTLIL	HLA-E*0101	
LC13	1	1MI5	DE	H	12530975	TRAV26-2*01	TRAJ52*01	TISG.....TDY	GLT.....SN	ILPLAGGTSYGKLT *	TRBV7-8*01	TRBJ2-7*01	SGH.......VS	FQN....EAQ	ASSLGQ..AYEQY	FLRGRAYGL	HLA-B*0801	
LC13	u	1KGC	DE	H	12429093	TRAV26-2*01	TRAJ52*01	TISG.....TDY	GLT.....SN	ILPLAGGTSYGKLT *	TRBV7-8*01	TRBJ2-7*01	SGH.......VS	FQN....EAQ	ASSLGQ..AYEQY			
LC13	1	3KPR	DE,IJ	H	20064448	TRAV26-2*01	TRAJ52*01	TISG.....TDY	GLT.....SN	ILPLAGGTSYGKLT *	TRBV7-8*01	TRBJ2-7*01	SGH.......VS	FQN....EAQ	ASSLGQ..AYEQY	EEYLKAWTF	HLA-B*4405	
LC13	1	3KPS	DE	H	20064448	TRAV26-2*01	TRAJ52*01	TISG.....TDY	GLT.....SN	ILPLAGGTSYGKLT *	TRBV7-8*01	TRBJ2-7*01	SGH.......VS	FQN....EAQ	ASSLGQ..AYEQY	EEYLQAFTY	HLA-B*4405	
MEL5	1	3HG1	DE	H	19605354	TRAV12-2*01	TRAJ27*01	DRG......SQS	IYS....NGD	AVNVA....GKST	TRBV30*01	TRBJ2-2*01	GTS......NPN	SVG.....IG	AWSETGLGTGELF	ELAGIGILTV	HLA-A*0201	
NKT12 [H]	u	2EYR	AB	H	16505140	TRAV10*01	TRAJ18*01	VSP......FSN	MTFS...ENT	VVSDRGSTLGRLY	TRBV25-1*01	TRBJ2-7*01	MGH.......DK	SYG....VNS	ASTSRR.GSYEQY			
iNKT [H,A]	u	2CDE	AB,CD,EF	H	16520393	TRAV10*01	TRAJ18*01	VSP......FSN	MTFS...ENT	VVSDRGSTLGRLY	TRBV25-1*01	TRBJ2-7*01	MGH.......DK	SYG....VNS	ASSENIGTAYEQY			
nkt15 [H]	1	3HUJ	EF,GH	H	19592275	TRAV10*01	TRAJ18*01	VSP......FSN	MTFS...ENT	VVSDRGSTLGRLY	TRBV25-1*01	TRBJ2-7*01	MGH.......DK	SYG....VNS	ASSGLRDRGLYEQY	CID 2826713	CD1d	
nkt15 [H]	1	2PO6	CD,GH	H	17581592	TRAV10*01	TRAJ18*01	VSP......FSN	MTFS...ENT	VVSDRGSTLGRLY	TRBV25-1*01	TRBJ2-7*01	MGH.......DK	SYG....VNS	ASSGLRDRGLYEQY	CID 2826713	CD1d	
nkt15 [H]	u	2EYS	AB	H	16505140	TRAV10*01	TRAJ18*01	VSP......FSN	MTFS...ENT	VVSDRGSTLGRLY	TRBV25-1*01	TRBJ2-7*01	MGH.......DK	SYG....VNS	ASSGLRDRGLYEQY			
nkt15 [H]	u	2EYT	AB,CD	H	16505140	TRAV10*01	TRAJ18*01	VSP......FSN	MTFS...ENT	VVSDRGSTLGRLY	TRBV25-1*01	TRBJ2-7*01	MGH.......DK	SYG....VNS	ASSGLRDRGLYEQY			
nkt 5B [H]	u	2CDG	AB	H	16520393	TRAV17*01	TRAJ18*01	TSI.......NN	IRSN...ERE	APFDRGSTLGRLY	TRBV25-1*01	TRBJ1-2*01	MGH.......DK	SYG....VNS	ASSESRTGINYGYT			
nkt 5E [H]	u	2CDF	AB	H	16520393	TRAV27*01	TRAJ18*01	SVF.......SS	VVTG...GEV	AGADRGSTLGRLY	TRBV25-1*01	TRBJ1-4*01	MGH.......DK	SYG....VNS	ASSEFRDGNEKLF			

a) Bound states: a=antibody; ; u =unbound, s=superantigen;1=MHC class I; 2=MHC class II
b) Differences to the IMGT AA sequence in CDR1/2 loops are underlined; differences of subtypes in the CDR3 loops are underlinded. (*) IMGTDomainGapAlign results were manually adjusted.
c) Peptides linked to MHC or TCR are marked with (L); For non-peptidic ligands the pubchem ID is given.
d) Y5 is mutated to K and linked to 4-(3-Indolyl)-butyric acid.
e) Mutation I219A in MHC α_3 domain
f) Mutation A245V in the MHC α3 domain.
g) Mutations Q65A, T69A, Q155A.
h) $H2\text{-}K1^{bm3}$ and $H2\text{-}K1^{bm8}$ are natural mutants of $H2\text{-}K1^{b}$
i) WT with solubility mutations.
j) Solubility Mutations (F9Y, V12T, I23T)
k) Mutation R189K
l) Mutation T163A in the MHC binding pocket
m) Mutation W167V in th MHC binding pocket
n) F5: 3,4-difluoro-phenylalanine.
o) F5: 4-fluoro-phenylalanine

S1 Table. Properties of available TCR structures (continued).

Name	Bound State[a]	PDB-ID	Chain Pairs	Spec.	Literature PMID	Alpha					Beta					Ligand		
						IMGT-Name		CDR Loops[b]			IMGT-Name		CDR-Loops[b]			Pep/Lip[c]	MHC I/II alpha	MHC II beta
						V-Segment	J-Segment	1	2	3	V-Segment	J-Segment	1	2	3			
OB.1A12	2	2WBJ	CD,GH	H	19303388	TRAV17*01	TRAJ40*01	TSI.......NN	IRSN...ERE	ATDTTS.GTYKYI	TRBV20-1*01	TRBJ2-1*01	DFQ......ATT	SNEG...SKA	SARDLTSGANNEQ	FARVHFISALHGS (L)	HLA-DRA*0101	HLA-DRB1*1501
OB.1A12	2	1YMM	DE	H	15821740	TRAV17*01	TRAJ40*01	TSI.......NN	IRSN...ERE	ATDTTS.GTYKYI	TRBV20-1*01	TRBJ2-1*01	DFQ......ATT	SNEG...SKA	SARDLTSGANNEQ	ENPVVHFFKNIVTPR (L)	HLA-DRA*0101	HLA-DRB1*1501
RA14	1	3GSN	AB	H	19542454	TRAV24*01	TRAJ49*01	SSN......FYA	MTLN...GDE	ARNTG....NQFY	TRBV6-5*01	TRBJ1-2*01	MNH.......EY	SVG....AGI	ASSPVTGGIYGYT	NLVPMVATV	HLA-A*0201 [f]	
SB27	1	2AK4	DE,IJ,NP,TU	H	16186824	TRAV19*01	TRAJ34*01	TRDT.....TYY	RNSF..DEQN	ALSGFY.NTDKLI	TRBV6-1*01	TRBJ2-7*01	MNH.......NS	SAS....EGT	ASPGLA.GEYEQY	LPEPLPQGQLTAY	HLA-B*3508	
SB27 [K16Dα]	1	3KXF	DE,GH,NP,MO	H	20483993	TRAV19*01	TRAJ34*01	TRDT.....TYY	RNSF..DEQN	ALSGFY.NTDKLI	TRBV6-1*01	TRBJ2-7*01	MNH.......NS	SAS....EGT	ASPGLA.GEYEQY	LPEPLPQGQLTAY	HLA-B*3508 [g]	
TCR MS2-3C8	2	3O6F	CD,GH	H	21297580	TRAV26-2*01	TRAJ32*01	TISG.....TDY	GLT.....SN	TVYGGA..TNKLI *	TRBV20-1*01	TRBJ1-6*01	DFQ......ATT	SNEG...SKA	SARGGS.YNSPLH	FSWGAEGQRPGFG (L)	HLA-DRA*0101	HLA-DRB1*0401
TK3 WT	1	3MV7	DE	H	PMC2901058	TRAV20*01	TRAJ52*01	VSG......LRG	LYSA...GEE	AVQDLGTSGSRLT *	TRBV9*01	TRBJ2-2*01	SGD.......LS	YYN....GEE	ASSAR...SGELF	HPVGEADYFEY	HLA-B*3501	
TK3 Q55H	1	3MV8	DE	H	PMC2901058	TRAV20*01	TRAJ52*01	VSG......LRG	LYSA...GEE	AVQDLGTSGSRLT *	TRBV9*02	TRBJ2-2*01	SGD.......LS	YYN....GEE	ASSAR...SGELF	HPVGEADYFEY	HLA-B*3501	
TK3 Q55A	1	3MV9	DE	H	PMC2901058	TRAV20*01	TRAJ52*01	VSG......LRG	LYSA...GEE	AVQDLGTSGSRLT *	TRBV9*01	TRBJ2-2*01	SGD.......LS	YYN....GEE	ASSAR...SGELF	HPVGEADYFEY	HLA-B*3501	
1934.4	2	2PXY	AB	M	17694060	TRAV6D-7*01	TRAJ48*01	ASG......YPA	ASRD...KEK	ALSENY.GNEKIT	TRBV13-2*01	TRBJ2-3*01	NNH.......NN	SYG....AGS	ASGDAS.GAETLY	HSRGGASQYRPSQ	H2-Aa(u)	H2-Ab(u)
1F1E8	u	3MFF	AB,CD	M	20630474	TRAV14D-3*2	TRAJ52*01	NSA......FDY	IRSV...SDK	AATGAN..TGKLT	TRBV29*01	TRBJ2-7*01	MSH.......ET	SYD....VDS	ASSLAGTGNYEQY			
226 TCR	2	3QIU	CD	M	21490152	TRAV4D-4*01	TRAJ16*01	TTM.......RA	LAS.....GT	AAEPSS..GQKLV	TRBV26*01	TRBJ1-2*01	KGH.......PV	FQN....QEV	ASSLNN.ANSDYT	ADLIAYLKQATKG	H2-Ea(k)	H2-Eb(k)
226 TCR	2	3QIW	CD	M	21490152	TRAV4D-4*01	TRAJ16*01	TTM.......RA	LAS.....GT	AAEPSS..GQKLV	TRBV26*01	TRBJ1-2*01	KGH.......PV	FQN....QEV	ASSLNN.ANSDYT	ADLIAYLEQATKG	H2-Ea(k)	H2-Eb(k)
2B4	1	3QIB	CD	M	21490152	TRAV4D-4*02	TRAJ56*01	TTM.......RA	LAS.....GT	AALRATGGNNKLT	TRBV26*01	TRBJ2-5*01	KGH.......PV	FQN....QEV	ASSLNW.SQDTQY	ADLIAYLKQATKG	H2-Ea(k)	H2-Eb(k)
2B4	u	3QJF	AB,CD	M	21490152	TRAV4D-4*02	TRAJ56*01	TTM.......RA	LAS.....GT	AALRATGGNNKLT	TRBV26*01	TRBJ2-5*01	KGH.......PV	FQN....QEV	ASSLNW.SQDTQY			
2C	u	1TCR	AB	M	8824178	TRAV9-4*01	TRAJ35*02	YSA......TPY	YYSG..DPVV	AVSGF...ASALT	TRBV13-2*01	TRBJ2-4*01	NNH.......NN	SYG....AGS	ASGGG....GTLY			
2C	1	1G6R	AB,CD	M	10755612	TRAV9-4*01	TRAJ35*02	YSA......TPY	YYSG..DPVV	AVSGF...ASALT	TRBV13-2*01	TRBJ2-4*01	NNH.......NN	SYG....AGS	ASGGG....GTLY	SIYRYYGL	H2-K1(b)	
2C	1	1MWA	AB, CD	M	11994422	TRAV9-4*01	TRAJ35*02	YSA......TPY	YYSG..DPVV	AVSGF...ASALT	TRBV13-2*01	TRBJ2-4*01	NNH.......NN	SYG....AGS	ASGGG....GTLY	EQYKFYSV	H2-K1(bm3) [h]	
2C	1	2CKB	AB,CD	M	9469799	TRAV9-4*01	TRAJ35*02	YSA......TPY	YYSG..DPVV	AVSGF...ASALT	TRBV13-2*01	TRBJ2-4*01	NNH.......NN	SYG....AGS	ASGGG....GTLY	EQYKFYSV	H2-K1(b)	
2C T7	S,2	2ICW	IJ,KL	M	17220897	TRAV9-4*01	TRAJ35*02	YSA......TPY	YYSG..DPVV	AVSGF...ASALT	TRBV13-2*01	TRBJ2-4*01	NNH.......NN	SYG....AGS	ASGGG....GTLY	PKYVKQNTLKLAT	HLA-DRA*0101	HLA-DRB1*0101
2C [T7-wt-s][i]	1	2OI9	BC	M	17418792	TRAV9-4*01	TRAJ35*02	YSA......TPY	YYSG..DPVV	AVSGF...ASALT	TRBV13-2*01	TRBJ2-4*01	NNH.......NN	SYG....AGS	ASGGG....GTLY	QLSPFPFDL	H2-L(d) [j]	
2C m13 [T7-s]	1	3E3Q	DE,CF,IJ,MN,RS,VW,Za,de	M	18941216	TRAV9-4*01	TRAJ35*02	YSA......TPY	YYSG..DPVV	AVSDP...PPLLT	TRBV13-2*01	TRBJ2-4*01	NNH.......NN	SYG....AGS	ASGGG....GTLY	QLSPFPFDL	H2-L(d) [j]	
2C m6 [T7-s]	1	2E7L	AD,BC	M	17418792	TRAV9-4*01	TRAJ35*02	YSA......TPY	YYSG..DPVV	AVSHQ...GRYLT	TRBV13-2*01	TRBJ2-4*01	NNH.......NN	SYG....AGS	ASGGG....GTLY	QLSPFPFDL	H2-L(d)	
2C m67 [T7-s]	1	3E2H	BC	M	18941216	TRAV9-4*01	TRAJ35*02	YSA......TPY	YYSG..DPVV	AVSLE...RPYLT	TRBV13-2*01	TRBJ2-4*01	NNH.......NN	SYG....AGS	ASGGG....GTLY	QLSPFPFDL	H2-L(d) [ij]	
2W20	2	3C6L	AB,EF	M	18308592	TRAV14-2*02	TRAJ31*01	DST......FDY	IRPV...SNK	AASD.....NRIF	TRBV13-2*01	TRBJ2-7*01	NNH.......NN	SYG....AGS	ASGDAW..GYEQY	FEAQKAKANKAVD (L)	H2-Aa(b)	H2-Ab(b) [k]
5c.c7	u	3QJH	AB,CD	M	21490152	TRAV4D-4*03	TRAJ34*02	TTM.......RA	LAS.....GT	AAEASN..TNKVV	TRBV26*01	TRBJ1-2*01	KGH.......PV	FQN....QEV	ASSLNN.ANSDYT			
AHIII12.2	1	2UWE	EF,LM	M	17825839	TRAV12D-2*01	TRAJ50*01	STY......SPF	SFTD...NKR	ALFLASSSFSKLV	TRBV13-3*01	TRBJ2-7*01	NNH.......DY	SYV....ADS	ASSDWV..SYEQY	ALWGFFPVL	HLA-A*0201 [l]	
AHIII12.2	1	2JCC	EF,LM	M	17825839	TRAV12D-2*01	TRAJ50*01	STY......SPF	SFTD...NKR	ALFLASSSFSKLV	TRBV13-3*01	TRBJ2-7*01	NNH.......DY	SYV....ADS	ASSDWV..SYEQY	ALWGFFPVL	HLA-A*0201 [m]	
AHIII12.2	1	1LP9	EF,LM	M	14563323	TRAV12D-2*01	TRAJ50*01	STY......SPF	SFTD...NKR	ALFLASSSFSKLV	TRBV13-3*01	TRBJ2-7*01	NNH.......DY	SYV....ADS	ASSDWV..SYEQY	ALWGFFPVL	HLA-A*0201	
B3K506	2	3C5Z	AB,EF	M	18308592	TRAV6-7*01	TRAJ34*02	ASG......YPA	ASRD...KEK	ALVISN..TNKVV	TRBV13-3*01	TRBJ1-3*01	NNH.......DY	SYV....ADS	ASIDSS..GNTLY	FEAQKAKANKAVD (L)	H2-Aa(b)	H2-Ab(b) [k]
BM3.3	1	1NAM	AB	M	12563259	TRAV16*01	TRAJ32*01	TQDS.....SYF	QDSY..KKEN	AMRGDYGGSGNKLI *	TRBV1*01	TRBJ1-3*01	NSQ......YPW	LRS....PGD	TCSADR.VGNTLY	RGYVYQGL	H2-K1(b)	
BM3.3	1	1FO0	AB	M	11017099	TRAV16*01	TRAJ32*01	TQDS.....SYF	QDSY..KKEN	AMRGDYGGSGNKLI *	TRBV1*01	TRBJ1-3*01	NSQ......YPW	LRS....PGD	TCSADR.VGNTLY	INFDFNTI	H2-K1(b)	
BM3.3	1	2OL3	AB	M	17363906	TRAV16*01	TRAJ32*01	TQDS.....SYF	QDSY..KKEN	AMRGDYGGSGNKLI *	TRBV1*01	TRBJ1-3*01	NSQ......YPW	LRS....PGD	TCSADR.VGNTLY	SQYYYNSL	H2-K1(bm8) [h]	
cl19	2	2Z31	AB	M	17694060	TRAV6D-7*01	TRAJ48*01	ASG......YPA	ASRD...KEK	ALSENY.GNEKIT	TRBV13-2*01	TRBJ2-4*01	NNH.......NN	SYG....AGS	ASGDAS.GGNTLY	RGGASQYRPSQ	H2-Aa(u)	H2-Ab(u)
D10	2	1D9K	AB,EF	M	10583947	TRAV14D-2*01	TRAJ11*01	DST......FDY	ISLV...SNK	AATGS...FNKLT	TRBV13-2*01	TRBJ2-1*01	NNH.......NN	SYG....AGS	ASGGQG..RAEQF	GNSHRGAIEWEGIESG	H2-Aa(k)	
KB5-C20	1	1KJ2	AB,DE	M	11911820	TRAV14-1*01	TRAJ15*01	EDST......FN	SIRSV...SD	CAARYQ.GGRALI	TRBV1*01	TRBJ2-3*01	NSQ......YPW	LRS....PGD	TCSAAPDWGASAE	KVITFIDL	H2-K1(b)	
N15	u,a	1NFD	AB,CD	M	9427737	TRAV12D-1*01	TRAJ40*01	TTYL.....TIA	SSTD...NKR	ALSEGG..NYKYV	TRBV12-1*01	TRBJ2-7*01	SGH.......SN	HYE....KVE	ASSLRW..GDEQY			
nkt [M,A]	1	3O8X	CD	M	20921281	TRAV11*02	TRAJ18*01	VTP......DNH	LVDQ...KDK	VVGDRGSALGRLH	TRBV13-2*01	TRBJ2-5*01	NNH.......NN	SYG....AGS	ASGDE...GYTQY	CID 6420173	CD1d1	
nkt [M,A]	1	3O9W	CD	M	20921281	TRAV11*02	TRAJ18*01	VTP......DNH	LVDQ...KDK	VVGDRGSALGRLH	TRBV13-2*01	TRBJ2-5*01	NNH.......NN	SYG....AGS	ASGDE...GYTQY	CID 44593851	CD1d1	
nkt [M,A]	1	3QUY	CD	M	21552205	TRAV11*02	TRAJ18*01	VTP......DNH	LVDQ...KDK	VVGDRGSALGRLH	TRBV13-2*01	TRBJ2-5*01	NNH.......NN	SYG....AGS	ASGDE...GYTQY	?	CD1d1	
nkt [M,A]	1	3QUX	CD	M	21552205	TRAV11*02	TRAJ18*01	VTP......DNH	LVDQ...KDK	VVGDRGSALGRLH	TRBV13-2*01	TRBJ2-5*01	NNH.......NN	SYG....AGS	ASGDE...GYTQY	CID 9811322	CD1d1	
Nkt [M,A]	1	3QUZ	CD	M	21552205	TRAV11*02	TRAJ18*01	VTP......DNH	LVDQ...KDK	VVGDRGSALGRLH	TRBV13-2*01	TRBJ2-5*01	NNH.......NN	SYG....AGS	ASGDE...GYTQY	CID 25228911	CD1d1	
nkt [M,B]	u	2Q86	AB,CD	M	18295796	TRAV11*02	TRAJ18*01	VTP......DNH	LVDQ...KDK	VVGDR....GSAL	TRBV13-2*01	TRBJ2-4*01	NNH.......NN	SYG....AGS	ASGGG....GTLY			
nkt [M,C]	1	3ARB	CD	M	21376639	TRAV11*02	TRAJ18*01	VTP......DNH	LVDQ...KDK	VVGDRGSALGRLH	TRBV13-2*01	TRBJ2-1*01	NNH.......NN	SYG....AGS	ASGDAGGNYAEQF	CID: 2826714	CD1d1	
nkt [M,C]	1	3ARD	CD	M	21376639	TRAV11*02	TRAJ18*01	VTP......DNH	LVDQ...KDK	VVGDRGSALGRLH	TRBV13-2*01	TRBJ2-1*01	NNH.......NN	SYG....AGS	ASGDAGGNYAEQF	CID 45273283)	CD1d1	
nkt [M,C]	1	3ARE	CD	M	21376639	TRAV11*02	TRAJ18*01	VTP......DNH	LVDQ...KDK	VVGDRGSALGRLH	TRBV13-2*01	TRBJ2-1*01	NNH.......NN	SYG....AGS	ASGDAGGNYAEQF	CID 45269857	CD1d1	
nkt [M,C]	1	3ARF	CD	M	21376639	TRAV11*02	TRAJ18*01	VTP......DNH	LVDQ...KDK	VVGDRGSALGRLH	TRBV13-2*01	TRBJ2-1*01	NNH.......NN	SYG....AGS	ASGDAGGNYAEQF	CID 50990900	CD1d1	
nkt [M,C]	1	3ARG	CD	M	21376639	TRAV11*02	TRAJ18*01	VTP......DNH	LVDQ...KDK	VVGDRGSALGRLH	TRBV13-2*01	TRBJ2-1*01	NNH.......NN	SYG....AGS	ASGDAGGNYAEQF	CID 50990901	CD1d1	
nkt [M,C]	1	3HE6	CD	M	19592275	TRAV11*02	TRAJ18*01	VTP......DNH	LVDQ...KDK	VVGDRGSALGRLH	TRBV13-2*01	TRBJ2-1*01	NNH.......NN	SYG....AGS	ASGDAGGNYAEQF	CID 2826713	CD1d1	
nkt [M,D]	1	3HE7	CD	M	19592275	TRAV11*02	TRAJ18*01	VTP......DNH	LVDQ...KDK	VVGDRGSALGRLH	TRBV29*01	TRBJ2-5*01	MSH.......ET	SYD....VDS	ASSSTG..LDTQY	CID 2826713	CD1d1	
TCR 21.30	2	3MBE	CD,GH	M	20407212	TRAV4-2*01	TRAJ32*01	TTM.......KS	LAQ.....GT	AAEDGG.SGNKLI	TRBV13-3*01	TRBJ1-3*01	NNH.......DY	SYV....ADS	ASSWDR.AGNTLY	GAMKRHGLDNYRGYSLGN	H2-Aa(d)	H2-Ab(NOD)
TCR172.10	2	1U3H	AB,EF	M	15664161	TRAV14D-3*08	TRAJ13*01	ENSA......FD	SILSV...SD	CAASAN.SGTYQR	TRBV13-2*01	TRBJ2-7*01	NNH.......NN	SYG....AGS	ASGDAG.GGYEQY	SRGGASQYRPSQ	H2-Aa(u)	H2-Ab(u)
YAe62	2	3C60	AB,EF	M	18308592	TRAV6-3*01	TRAJ13*01	TTG......YPT	VTTA...NNK	AANSG....TYQR	TRBV13-2*01	TRBJ2-4*01	NNH.......NN	SYG....AGS	ASGDFW..GDTLY	FEAQKAKANKAVD (L)	H2-Aa(b)	H2-Ab(b) [k]

a) Bound states: a=antibody; ; u =unbound, s=superantigen;1=MHC class I; 2=MHC class II

b) Differences to the IMGT AA sequence in CDR1/2 loops are underlined; differences of subtypes in the CDR3 loops are underlinded. (*) IMGTDomainGapAlign results were manually adjusted.

c) Peptides linked to MHC or TCR are marked with (L); For non-peptidic ligands the pubchem ID is given.

d) Y5 is mutated to K and linked to 4-(3-Indolyl)-butyric acid.

e) Mutation I219A in MHC α_3 domain

f) Mutation A245V in the MHC α3 domain.

g) Mutations Q65A, T69A, Q155A.

h) H2-K1^{bm3} and H2-K1^{bm8} are natural mutants of H2-K1^{b}

i) WT with solubility mutations.

j) Solubility Mutations (F9Y, V12T, I23T)

k) Mutation R189K

l) Mutation T163A in the MHC binding pocket

m) Mutation W167V in th MHC binding pocket

n) F5: 3,4-difluoro-phenylalanine.

o) F5: 4-fluoro-phenylalanine

1. Giudicelli V, Chaume D, Lefranc M-P (2005) IMGT/GENE-DB: a comprehensive database for human and mouse immunoglobulin and T cell receptor genes. Nucleic Acids Res 33: D256-D261.
