# Supplementary material for: Quantitative Analysis of the Association Angle between T-cell Receptor Vα/Vβ Domains Reveals Important Features for Epitope Recognition
Source: PLoS Comput Biol. 2015 Jul 17;11(7):e1004244. doi: 10.1371/journal.pcbi.1004244 (PMC4505886; doi:10.1371/journal.pcbi.1004244)
Supplement: S2 Table — The sequences of the TCR structures were compared to the corresponding wild type (WT). Differences to the alleles provided in the IMGT Gene-DB [61] are provided as mutation pairs in brackets. Naming of the sheets and loops, and the residue indices follow the IMGT unique numbering [62]. (PDF) [file pcbi.1004244.s003.pdf]

S2 Table. Mutations within the framework region of the TCR structures. The sequences of the TCR structures were compared to the corresponding wild type (WT). Differences to the alleles provided in the IMGT Gene-DB [1] are provided as mutation pairs in brackets. Naming of the sheets and loops, and the residue indices follow the IMGT unique numbering [2].

Name	PDB-ID	Alpha Chain Mutations in Respect To WT Sequence Acc. To IMGT Unique Numbering (w/o CDR3/FG)											Beta Chain Mutations in Respect To WT Sequence Acc. To IMGT Unique Numbering (w/o CDR3/FG)										
		A	B	BC	C	C'	C'C''	C''	D	E	F	G	A	B	BC	C	C'	C'C''	C''	D	E	F	G
1G4 AV-wt	2F54	(E3Q)				(K48G)(G49K)			(S84A)	(R86S)													
1G4 c5c1	2PYF									(R86S)								(I65T)		(T84I)			
1G4 c5c1	2PYE									(R86S)								(I65T)		(T84I)			
1G4 c49c50	2F53						(Q57P)(S58F)(S59W)											(G58S)(A63V)(I65M)					
1G4 c58c62	2P5W	(E3Q)					(Q57T)(S58P)(S59W)			(R86S)								(G58S)(A63V)(I65M)		(T84I)			
1G4 c58c61	2P5E						(Q57T)(S58P)(S59W)			(R86S)								(G58A)(A63I)(G64Q)(I65T)		(T84I)			
3A6	1ZGL																	(S58N)					
AS01	3O4L								(L76K)														
B7	1BD2							(E67A)															
cf34	3FFC					(L53V)	(Q64G)																
DM1	3DXA	(P9T)																					
DM1	3DX9	(P9T)																					
Hy.1B1	3PL6											(A120T)(K123R)(K127I)											
MEL5	3HG1	(K2Q)																					
nkt 5E [H]	2CDF	(L4V)																					
OB.1A12	2WBJ												(C13S)										
OB.1A12	1YMM												(C13S)										
SB27 [K16Dα]	3KXF		(K16D)																				
TCR MS2-3C8	3O6F												(C13A)										
TK3 WT	3MV7											(Q120E)(I123Q)(H128N)											
TK3 Q55H	3MV8											(Q120E)(I123Q)(H128N)					(Q55H)						
TK3 Q55A	3MV9											(Q120E)(I123Q)(H128N)					(Q55A)						
1934,4	2PXY	(E8G)										(T125Q)(I126V)(K127V)		(G17E)			(H54Y)				(I90T)(L93S)		(S120P)
1F1E8	3MFF		(A19T)																				
2C T7	2ICW					(L50P)				(W96R)				(G17E)			(G49E)				(L93S)		
2C [T7-wt-s]	2OI9					(L50P)				(W96R)				(G17E)			(G49E)(H54Y)				(I90T)(L93S)		
2C m13 [T7-s]	3E3Q					(L50P)				(W96R)				(G17E)			(G49E)(H54Y)				(I90T)(L93S)		
2C m6 [T7-s]	2E7L					(L50P)				(W96R)				(G17E)			(G49E)(H54Y)				(I90T)(L93S)		
2C m67 [T7-s]	3E2H					(L50P)				(W96R)				(G17E)			(G49E)(H54Y)				(I90T)(L93S)		
B3K506	3C5Z	(Q10N)																					
BM3.3	1FO0											(I120T)											
BM3.3	2OL3											(I120T)											
cl19	2Z31	(E8G)										(T125Q)(I126V)(K127V)		(G17E)			(H54Y)				(I90T)(L93S)		
D10	1D9K											(K120A)(V123R)(L125A)											
nkt [M,B]	2Q86				(F42Y)	(S52L)					(I103F)	(A120S)											
nkt [M,C]	3ARB		(C19S)																				
nkt [M,C]	3ARD		(C19S)																				
nkt [M,C]	3ARE		(C19S)																				
nkt [M,C]	3ARF		(C19S)																				
nkt [M,C]	3ARG		(C19S)																				
nkt [M,C]	3HE6		(C19S)																				
nkt [M,D]	3HE7		(C19S)																				
TCR 21.30	3MBE											(I120T)											
TCR172.10	1U3H													(G17E)			(H54Y)				(I90T)(L93S)		
YAe62	3C60	(I5T)																					

1. Giudicelli V, Chaume D, Lefranc M-P (2005) IMGT/GENE-DB: a comprehensive database for human and mouse immunoglobulin and T cell receptor genes. Nucleic Acids Res 33: D256-D261.
2. Lefranc MP, Pommie C, Ruiz M, Giudicelli V, Foulquier E, et al. (2003) IMGT unique numbering for immunoglobulin and T cell receptor variable domains and Ig superfamily V-like domains. Dev Comp Immunol 27: 55-77.
