# Supplementary material for: Quantitative Analysis of the Association Angle between T-cell Receptor Vα/Vβ Domains Reveals Important Features for Epitope Recognition
Source: PLoS Comput Biol. 2015 Jul 17;11(7):e1004244. doi: 10.1371/journal.pcbi.1004244 (PMC4505886; doi:10.1371/journal.pcbi.1004244)
Supplement: S3 Table — (PDF) [file pcbi.1004244.s004.pdf]

S3 Table. Multiple Sequence Alignments at the COR.

C	Name	PDB	COR α	COR β
1	1G4 c49c50	2f53	lqwfrQdpgkg	mswyrQdpgmg
	1G4	2bnr	lqwfrQdpgkg	mswyrQdpgmg
	1G4	2bnq	lqwfrQdpgkg	mswyrQdpgmg
	1G4 AV-wt	2f54	lqwfrQdpggk	mswyrQdpgmg
	E8	2ian	lqwfhQnpwgq	mywyrQdpgmg
	1G4 c58c61	2p5e	lqwfrQdpgkg	mswyrQdpgmg
	1G4 c58c62	2p5w	lqwfrQdpgkg	mswyrQdpgmg
	E8	2iam	lqwfhQnpwgq	mywyrQdpgmg
	1G4 c5c1	2pye	lqwfrQdpgkg	mswyrQdpgmg
2	TCR MS2-3C8	3o6f	ihwyrQlpsqg	mfwyrQfpkqs
	A6	1qse	ffwyrQysgks	mswyrQdpgmg
	A6	3h9s	ffwyrQysgks	mswyrQdpgmg
	A6	3d39	ffwyrQysgks	mswyrQdpgmg
	A6	3d3v	ffwyrQysgks	mswyrQdpgmg
	A6	3pwp	ffwyrQysgks	mswyrQdpgmg
	A6	1qsf	ffwyrQysgks	mswyrQdpgmg
	A6	2gj6	ffwyrQysgks	mswyrQdpgmg
	A6	1ao7	ffwyrQysgks	mswyrQdpgmg
	A6	1qrn	ffwyrQysgks	mswyrQdpgmg
3	JM22	2vlj	lqwyrQepgeg	mywyrQdpgqg
	JM22	2vlk	lqwyrQepgeg	mywyrQdpgqg
	JM22	1oga	lqwyrQepgeg	mywyrQdpgqg
	JM22 [S99βA]	2vlr	lqwyrQepgeg	mywyrQdpgqg
	SB27	2ak4	lfwykQppsge	mywyrQdpgmg
	SB27 [K16Dα]	3kxf	lfwykQppsge	mywyrQdpgmg
	TCR 21.30	3mbe	vqwfqQnhrgr	mywyrQdtghg
	OB.1A12	1ymm	lqwyrQnsgrg	mfwyrQfpkqs
	OB.1A12	2wbj	lqwyrQnsgrg	mfwyrQfpkqs
4	KK50.4	2esv	vywyrQihsqg	lywyrRvmgke
	LC13	3kpr	ihwyrQlpsqg	lfwyqQalgqg
	2B4	3qib	vqwfqQnsrgs	vfwyqQnknne
	LC13	3kps	ihwyrQlpsqg	lfwyqQalgqg
	226 TCR	3qiu	vqwfrKnsrgs	vfwyqQnknne
	226 TCR	3qiw	vqwfrKnsrgs	vfwyqQnknne
	2C m13 [T7-s]	3e3q	lfwyvQyprqg	mywyrQdtghe
	2C m6 [T7-s]	2e7l	lfwyvQyprqg	mywyrQdtghe
	2C [T7-wt-s]	2oi9	lfwyvQyprqg	mywyrQdtghe
	DM1	3dxa	vywyrQihsqg	lywyrQtlgqg
	LC13	1mi5	ihwyrQlpsqg	lfwyqQalgqg
	cf34	3ffc	lfwykQpssge	lywyqQilgqg
	BM3.3	2ol3	lfwykQtasge	mswyqQdlqkq
	BM3.3	1fo0	lfwykQtasge	mswyqQdlqkq
	BM3.3	1nam	lfwykQtasge	mswyqQdlqkq
5	D10	1d9k	fpwyrQfpgks	mywyrQdtghg
	2W20	3c6l	fpwyhQfpges	mywyrQdtghg
	AS01	3o4l	lywykQepgag	mfwyrQfpkqs
	KB5-C20	1kj2	fpwyqQfpgeg	mswyqQdlqkq
	HA1.7	1fyt	lfwyvQypnqg	mfwyrQdpglg
	HA1.7	1j8h	lfwyvQypnqg	mfwyrQdpglg
6	AHIII12.2	2jcc	lfwyvQhlnea	mywyrQdtghg
	AHIII12.2	2uwe	lfwyvQhlnea	mywyrQdtghg
	1934.4	2pxy	lfwyvQypgeg	mywyrQdtghg
	AHIII12.2	1lp9	lfwyvQhlnea	mywyrQdtghg

cl19	2z31	lfwyvQypgeg	mywyrQdtghg
B7	1bd2	flwykKypaeg	mswyrQdpgmg
TCR172.10	1u3h	fpwyqQfpgeg	mywyrQdtghg
B3K506	3c5z	lfwyvQypgeg	mywyrQdtghg
ELS4	2nx5	lfwyqQhagea	mywyrQdpghg
YAe62	3c60	lfwyvQysgeg	mywyrQdtghg
2C	2ckb	lfwyvQyprqg	mywyrQdtghg
2C	1mwa	lfwyvQyprqg	mywyrQdtghg
RA14	3gsn	lhwyrWetaks	mswyrQdpgmg
2C	1g6r	lfwyvQyprqg	mywyrQdtghg
Hy.1B1	3pl6	fpwykQelgkr	lywyrQslgqg
TK3 WT	3mv7	lfwyrQdpgkg	vywyqQsldqg
TK3 Q55H	3mv8	lfwyrQdpgkg	vywyqQsldqg
TK3 Q55A	3mv9	lfwyrQdpgkg	vywyqQsldqg
3A6	1zgl	lfwyvQypgeg	vswyqQtpgqg
2C m67 [T7-s]	3e2h	lfwyvQyprqg	mywyrQdtghe
2C T7	2icw	lfwyvQyprqg	mywyrQdtghe
MEL5	3hg1	ffwyrQysgks	lywyrQaagrg
