# Supplementary material for: Quantitative Analysis of the Association Angle between T-cell Receptor Vα/Vβ Domains Reveals Important Features for Epitope Recognition
Source: PLoS Comput Biol. 2015 Jul 17;11(7):e1004244. doi: 10.1371/journal.pcbi.1004244 (PMC4505886; doi:10.1371/journal.pcbi.1004244)
Supplement: S4 Table — (PDF) [file pcbi.1004244.s005.pdf]

S4 Table. Epitopes of the bound TCR structures.

C	Name	PDB	Peptide	MHC I/II α	MHC II β
1	1G4 c49c50	2f53	SLLMWITQC	HLA-A*0201	
	1G4	2bnr	SLLMWITQC	HLA-A*0201	
	1G4	2bnq	SLLMWITQV	HLA-A*0201	
	1G4 AV-wt	2f54	SLLMWITQC	HLA-A*0201	
	E8	2ian	GELIGTLNAAKVPAD	HLA-DRA*0101	HLA-DRB1*0101
	1G4 c58c61	2p5e	SLLMWITQC	HLA-A*0201	
	1G4 c58c62	2p5w	SLLMWITQC	HLA-A*0201	
	E8	2iam	GELIGILNAAKVPAD	HLA-DRA*0101	HLA-DRB1*0101
	1G4 c5c1	2pye	SLLMWITQC	HLA-A*0201	
2	TCR MS2-3C8	3o6f	FSWGAEGQRPGFG	HLA-DRA*0101	HLA-DRB1*0401
	A6	1qse	LLFGYPRYV	HLA-A*0201	
	A6	3h9s	MLWGYLQYV	HLA-A*0201	
	A6	3d39	LLFGFPVYV[k]	HLA-A*0201	
	A6	3d3v	LLFGFPVYV[l]	HLA-A*0201	
	A6	3pwp	LGYGFVNYI	HLA-A*0201	
	A6	1qsf	LLFGYPVAV	HLA-A*0201[a]	
	A6	2gj6	LLFGKPVYV[b]	HLA-A*0201	
	A6	1ao7	LLFGYPVYV	HLA-A*0201	
	A6	1qrn	LLFGYAVYV	HLA-A*0201	
3	JM22	2vlj	GILGFVFTL	HLA-A*0201	
	JM22	2vlk	GILGFVFTL	HLA-A*0201	
	JM22	1oga	GILGFVFTL	HLA-A*0201	
	JM22 [S99βA]	2vlr	GILGFVFTL	HLA-A*0201	
	SB27	2ak4	LPEPLPQGQLTAY	HLA-B*3508	
	SB27 [K16Dα]	3kxf	LPEPLPQGQLTAY	HLA-B*3508[c]	
	TCR 21.30	3mbe	GAMKRHGLDNYRGYSLGN	H2-Aa(d)	H2-Ab(NOD)
	OB.1A12	1ymm	ENPVVHFFKNIVTPR	HLA-DRA*0101	HLA-DRB1*1501
	OB.1A12	2wbj	FARVHFISALHGS	HLA-DRA*0101	HLA-DRB1*1501
4	KK50.4	2esv	VMAPRTLIL	HLA-E*0101	
	LC13	3kpr	EEYLKAWTF	HLA-B*4405	
	2B4	3qib	ADLIAYLKQATKG	H2-Ea(k)	H2-Eb(k)
	LC13	3kps	EEYLQAFTY	HLA-B*4405	
	226 TCR	3qiu	ADLIAYLKQATKG	H2-Ea(k)	H2-Eb(k)
	226 TCR	3qiw	ADLIAYLEQATKG	H2-Ea(k)	H2-Eb(k)
	2C m13 [T7-s]	3e3q	QLSPFPFDL	H2-L(d)[d]	
	2C m6 [T7-s]	2e7l	QLSPFPFDL	H2-L(d)	
	2C [T7-wt-s]	2oi9	QLSPFPFDL	H2-L(d)[d]	
	DM1	3dxa	EENLLDFVRF	HLA-B*4405	
	LC13	1mi5	FLRGRAYGL	HLA-B*0801	
	cf34	3ffc	FLRGRAYGL	HLA-B*0801	
	BM3.3	2ol3	SQYYYNSL	H2-K1(bm8)[e]	
	BM3.3	1fo0	INFDFNTI	H2-K1(b)	
	BM3.3	1nam	RGYVYQGL	H2-K1(b)	
5	D10	1d9k	GNSHRGAIEWEGIESG	H2-Aa(k)	H2-Ab(k)
	2W20	3c6l	FEAQKAKANKAVD	H2-Aa(b)	H2-Ab(b)[f]
	AS01	3o4l	GLCTLVAML	HLA-A*0201	
	KB5-C20	1kj2	KVITFIDL	H2-K1(b)	
	HA1.7	1fyt	PKYVKQNTLKLAT	HLA-DRA*0101	HLA-DRB1*0101
	HA1.7	1j8h	PKYVKQNTLKLAT	HLA-DRA*0101	HLA-DRB1*0401[j]
6	AHIII12.2	2jcc	ALWGFFPVL	HLA-A*0201[g]	
	AHIII12.2	2uwe	ALWGFFPVL	HLA-A*0201[h]	
	1934.4	2pxy	HSRGGASQYRPSQ	H2-Aa(u)	H2-Ab(u)
	AHIII12.2	1lp9	ALWGFFPVL	HLA-A*0201	
	cl19	2z31	RGGASQYRPSQ	H2-Aa(u)	H2-Ab(u)
	B7	1bd2	LLFGYPVYV	HLA-A*0201	
	TCR172.10	1u3h	SRGGASQYRPSQ	H2-Aa(u)	H2-Ab(u)
	B3K506	3c5z	FEAQKAKANKAVD	H2-Aa(b)	H2-Ab(b)[f]
	ELS4	2nx5	EPLPQGQLTAY	HLA-B*3501	

YAe62	3c60	FEAQKAKANKAVD	H2-Aa(b)	H2-Ab(b)[f]
2C	2ckb	EQYKFYSV	H2-K1(b)	
2C	1mwa	EQYKFYSV	H2-K1(bm3)[e]	
RA14	3gsn	NLVPMVATV	HLA-A*0201[i]	
2C	1g6r	SIYRYYGL	H2-K1(b)	
Hy.1B1	3pl6	NPVVHFFKNIVTPR	HLA-DQA1*0102	HLA-DQB1*0501
TK3 WT	3mv7	HPVGEADYFEY	HLA-B*3501	
TK3 Q55H	3mv8	HPVGEADYFEY	HLA-B*3501	
TK3 Q55A	3mv9	HPVGEADYFEY	HLA-B*3501	
3A6	1zgl	VHFFKNIVTPRTP	HLA-DRA*0101	HLA-DRB5*0101
2C m67	3e2h	QLSPFPFDL	H2-L(d)[d]	
(2C T7	2icw	PKYVKQNTLKLAT	HLA-DRA*0101	HLA-DRB1*0101)[s]
MEL5	3hg1	ELAGIGILTV	HLA-A*0201	
(JM22	2xn9	PKYVKQNTLKLAT	HLA-DRA*0101	HLA-DRB1*0101)[s]

Haplotypes of murine MHC H2 alleles are given in brackets to avoid confusion with footnotes.
a) Mutation I219A in MHC a_3 domain.
b) Y5 is mutated to K and linked to 4-(3-Indolyl)-butyric acid.
c) Mutation Q65A, T69A, Q155A
d) Solubility Mutations (F9Y, V12T, I23T)
e) H2-K1^{bm3} and H2-K1^{bm8} are natural mutants of H2-K1^{b}
f) Mutation R189K
g) Mutation W167V in the MHC binding pocket
h) Mutation T163A in the MHC binding pocket
i) Mutation A245V in the MHC a_3 domain
j) Mutation L209V in the MHC a_3 domain
k) Flouration: y5f^{4F}
l) Double-fluoration: y5f^{3,4FF}
s) Bound to super-antigen (Sag). The TCRs only directly contacts the SAgs and thus are not considered as MHC bound within the clustering process. However, the structures would associate with the cluster 6.
