# Supplementary material for: Quantitative Analysis of the Association Angle between T-cell Receptor Vα/Vβ Domains Reveals Important Features for Epitope Recognition
Source: PLoS Comput Biol. 2015 Jul 17;11(7):e1004244. doi: 10.1371/journal.pcbi.1004244 (PMC4505886; doi:10.1371/journal.pcbi.1004244)
Supplement: S5 Table — (PDF) [file pcbi.1004244.s006.pdf]

S5 Table. References to the TCR structures used for the analysis

Name	PDB	Ref.	Name	PDB	Ref.
1G4	2bnu	[1]	LC13	3kps	[2]
1G4	2bnq	[1]	MEL5	3hg1	[3]
1G4	2bnr	[1]	OB.1A12	2wbj	[4]
1G4 AV-wt	2f54	[5]	OB.1A12	1ymm	[6]
1G4 c5c1	2pyf	[7]	RA14	3gsn	[8]
1G4 c5c1	2pye	[7]	SB27	2ak4	[9]
1G4 c49c50	2f53	[5]	SB27[K16Dα]	3kxf	[10]
1G4 c58c62	2p5w	[7]	TCR MS2-3C8	3o6f	[11]
1G4 c58c61	2p5e	[7]	TK3 wt	3mv7	[12]
3A6	1zgl	[13]	TK3 Q55H	3mv8	[12]
A6	2gj6	[14]	TK3 Q55A	3mv9	[12]
A6	1qsf	[15]	1934,4	2pxy	[16]
A6	1qse	[15]	1F1E8	3mff	[17]
A6	3d3v	[18]	226 TCR	3qiu	[19]
A6	3d39	[18]	226 TCR	3qiw	[19]
A6	1qrn	[15]	2B4	3qib	[19]
A6	1ao7	[20]	2B4	3qjf	[19]
A6	3h9s	[21]	2C	1tcr	[22]
A6	3pwp	[23]	2C	1g6r	[24]
AS01	3o4l	[25]	2C	1mwa	[26]
B7	1bd2	[27]	2C	2ckb	[28]
cf34	3ffc	[29]	2C T7	2icw	[30]
DM1	3dxa	[31]	2C [T7-wt-s][a]	2oi9	[32]
DM1	3dx9	[31]	2C m13 [T7-s][a]	3e3q	[33]
E8	2ian	[34]	2C m6 [T7-s][a]	2e7l	[32]
E8	2iam	[34]	2C m67 [T7-s][a]	3e2h	[33]
E8	2ial	[34]	2W20	3c6l	[35]
ELS4	2nx5	[36]	5c.c7	3qjh	[19]
ELS4	2nw2	[36]	AHIII12.2	2uwe	[37]
HA1.7	1fyt	[38]	AHIII12.2	2jcc	[37]
HA1.7	1j8h	[39]	AHIII12.2	1lp9	[40]
Hy.1B1	3pl6	[41]	B3K506	3c5z	[35]
JM22	2vlj	[42]	BM3.3	1nam	[43]
JM22	2vlk	[42]	BM3.3	1fo0	[44]
JM22	1oga	[45]	BM3.3	2ol3	[46]
JM22	2vlm	[42]	cl19	2z31	[16]
JM22	2xn9	[47]	D10	1d9k	[48]
JM22	2xna	[47]	KB5-C20	1kj2	[49]
JM22 [S99βA]	2vlr	[42]	N15	1nfd	[50]
KK50.4	2esv	[51]	TCR 21.30	3mbe	[52]
LC13	1mi5	[53]	TCR172.10	1u3h	[54]
LC13	1kgc	[55]	YAe62	3c60	[35]
LC13	3kpr	[2]			

[a] WT with solubility mutations acc. to ref. [32]. More detailed information is available in Table S2.

SI References:

1. Chen J-L, Stewart-Jones G, Bossi G, Lissin NM, Wooldridge L, et al. (2005) Structural and kinetic basis for heightened immunogenicity of T cell vaccines. J Exp Med 201: 1243-1255.
2. Macdonald WA, Chen Z, Gras S, Archbold JK, Tynan FE, et al. (2009) T cell allorecognition via molecular mimicry. Immunity 31: 897-908.
3. Cole DK, Yuan F, Rizkallah PJ, Miles JJ, Gostick E, et al. (2009) Germ line-governed recognition of a cancer epitope by an immunodominant human T-cell receptor. J Biol Chem 284: 27281-27289.
4. Harkiolaki M, Holmes SL, Svendsen P, Gregersen JW, Jensen LT, et al. (2009) T cell-mediated autoimmune disease due to low-affinity crossreactivity to common microbial peptides. Immunity 30: 348-357.
5. Dunn SM, Rizkallah PJ, Baston E, Mahon T, Cameron B, et al. (2006) Directed evolution of human T cell receptor CDR2 residues by phage display dramatically enhances affinity for cognate peptide-MHC without increasing apparent cross-reactivity. Protein Sci 15: 710-721.
6. Hahn M, Nicholson MJ, Pyrdol J, Wucherpfennig KW (2005) Unconventional topology of self peptide-major histocompatibility complex binding by a human autoimmune T cell receptor. Nat Immunol 6: 490-496.
7. Sami M, Rizkallah PJ, Dunn S, Molloy P, Moysey R, et al. (2007) Crystal structures of high affinity human T-cell receptors bound to peptide major histocompatibility complex reveal native diagonal binding geometry. Protein Eng Des Sel 20: 397-403.
8. Gras S, Saulquin X, Reiser J-B, Debeaupuis E, Echasserieau K, et al. (2009) Structural bases for the affinity-driven selection of a public TCR against a dominant human cytomegalovirus epitope. J Immunol 183: 430-437.
9. Tynan FE, Burrows SR, Buckle AM, Clements CS, Borg NA, et al. (2005) T cell receptor recognition of a 'super-bulged' major histocompatibility complex class I-bound peptide. Nat Immunol 6: 1114-1122.
10. Burrows SR, Chen Z, Archbold JK, Tynan FE, Beddoe T, et al. (2010) Hard wiring of T cell receptor specificity for the major histocompatibility complex is underpinned by TCR adaptability. Proc Natl Acad Sci U S A 107: 10608-10613.
11. Yin Y, Li Y, Kerzic MC, Martin R, Mariuzza RA (2011) Structure of a TCR with high affinity for self-antigen reveals basis for escape from negative selection. EMBO J 30: 1137-1148.
12. Gras S, Chen Z, Miles JJ, Liu YC, Bell MJ, et al. (2010) Allelic polymorphism in the T cell receptor and its impact on immune responses. J Exp Med 207: 1555-1567.
13. Li Y, Huang Y, Lue J, Quandt JA, Martin R, et al. (2005) Structure of a human autoimmune TCR bound to a myelin basic protein self-peptide and a multiple sclerosis-associated MHC class II molecule. EMBO J 24: 2968-2979.
14. Gagnon SJ, Borbulevych OY, Davis-Harrison RL, Turner RV, Damirjian M, et al. (2006) T cell receptor recognition via cooperative conformational plasticity. J Mol Biol 363: 228-243.
15. Ding YH, Baker BM, Garboczi DN, Biddison WE, Wiley DC (1999) Four A6-TCR/peptide/HLA-A2 structures that generate very different T cell signals are nearly identical. Immunity 11: 45-56.

16. Feng D, Bond CJ, Ely LK, Maynard J, Garcia KC (2007) Structural evidence for a germline-encoded T cell receptor-major histocompatibility complex interaction 'codon'. Nat Immunol 8: 975-983.
17. van Boxel GI, Holmes S, Fugger L, Jones EY (2010) An alternative conformation of the T-cell receptor alpha constant region. J Mol Biol 400: 828-837.
18. Piepenbrink KH, Borbulevych OY, Sommese RF, Clemens J, Armstrong KM, et al. (2009) Fluorine substitutions in an antigenic peptide selectively modulate T-cell receptor binding in a minimally perturbing manner. Biochem J 423: 353-361.
19. Newell EW, Ely LK, Kruse AC, Reay PA, Rodriguez SN, et al. (2011) Structural basis of specificity and cross-reactivity in T cell receptors specific for cytochrome c-I-E(k). J Immunol 186: 5823-5832.
20. Garboczi DN, Ghosh P, Utz U, Fan QR, Biddison WE, et al. (1996) Structure of the complex between human T-cell receptor, viral peptide and HLA-A2. Nature 384: 134-141.
21. Borbulevych OY, Piepenbrink KH, Gloor BE, Scott DR, Sommese RF, et al. (2009) T cell receptor cross-reactivity directed by antigen-dependent tuning of peptide-MHC molecular flexibility. Immunity 31: 885-896.
22. Garcia KC, Degano M, Stanfield RL, Brunmark A, Jackson MR, et al. (1996) An alphabeta T cell receptor structure at 2.5 A and its orientation in the TCR-MHC complex. Science 274: 209-219.
23. Borbulevych OY, Piepenbrink KH, Baker BM (2011) Conformational melding permits a conserved binding geometry in TCR recognition of foreign and self molecular mimics. J Immunol 186: 2950-2958.
24. Degano M, Garcia KC, Apostolopoulos V, Rudolph MG, Teyton L, et al. (2000) A functional hot spot for antigen recognition in a superagonist TCR/MHC complex. Immunity 12: 251-261.
25. Miles JJ, Bulek AM, Cole DK, Gostick E, Schauenburg AJA, et al. (2010) Genetic and structural basis for selection of a ubiquitous T cell receptor deployed in Epstein-Barr virus infection. PLoS Pathog 6: e1001198.
26. Luz JG, Huang M, Garcia KC, Rudolph MG, Apostolopoulos V, et al. (2002) Structural comparison of allogeneic and syngeneic T cell receptor-peptide-major histocompatibility complex complexes: a buried alloreactive mutation subtly alters peptide presentation substantially increasing V(beta) Interactions. J Exp Med 195: 1175-1186.
27. Ding YH, Smith KJ, Garboczi DN, Utz U, Biddison WE, et al. (1998) Two human T cell receptors bind in a similar diagonal mode to the HLA-A2/Tax peptide complex using different TCR amino acids. Immunity 8: 403-411.
28. Garcia KC, Degano M, Pease LR, Huang M, Peterson PA, et al. (1998) Structural basis of plasticity in T cell receptor recognition of a self peptide-MHC antigen. Science 279: 1166-1172.
29. Gras S, Burrows SR, Kjer-Nielsen L, Clements CS, Liu YC, et al. (2009) The shaping of T cell receptor recognition by self-tolerance. Immunity 30: 193-203.
30. Wang L, Zhao Y, Li Z, Guo Y, Jones LL, et al. (2007) Crystal structure of a complete ternary complex of TCR, superantigen and peptide-MHC. Nat Struct Mol Biol 14: 169-171.
31. Archbold JK, Macdonald WA, Gras S, Ely LK, Miles JJ, et al. (2009) Natural micropolymorphism in human leukocyte antigens provides a basis for genetic control of antigen recognition. J Exp Med 206: 209-219.
32. Colf LA, Bankovich AJ, Hanick NA, Bowerman NA, Jones LL, et al. (2007) How a single T cell receptor recognizes both self and foreign MHC. Cell 129: 135-146.

33. Jones LL, Colf LA, Stone JD, Garcia KC, Kranz DM (2008) Distinct CDR3 conformations in TCRs determine the level of cross-reactivity for diverse antigens, but not the docking orientation. J Immunol 181: 6255-6264.
34. Deng L, Langley RJ, Brown PH, Xu G, Teng L, et al. (2007) Structural basis for the recognition of mutant self by a tumor-specific, MHC class II-restricted T cell receptor. Nat Immunol 8: 398-408.
35. Dai S, Huseby ES, Rubtsova K, Scott-Browne J, Crawford F, et al. (2008) Crossreactive T Cells spotlight the germline rules for alphabeta T cell-receptor interactions with MHC molecules. Immunity 28: 324-334.
36. Tynan FE, Reid HH, Kjer-Nielsen L, Miles JJ, Wilce MCJ, et al. (2007) A T cell receptor flattens a bulged antigenic peptide presented by a major histocompatibility complex class I molecule. Nat Immunol 8: 268-276.
37. Miller PJ, Pazy Y, Conti B, Riddle D, Appella E, et al. (2007) Single MHC mutation eliminates enthalpy associated with T cell receptor binding. J Mol Biol 373: 315-327.
38. Hennecke J, Carfi A, Wiley DC (2000) Structure of a covalently stabilized complex of a human alphabeta T-cell receptor, influenza HA peptide and MHC class II molecule, HLA-DR1. EMBO J 19: 5611-5624.
39. Hennecke J, Wiley DC (2002) Structure of a complex of the human alpha/beta T cell receptor (TCR) HA1.7, influenza hemagglutinin peptide, and major histocompatibility complex class II molecule, HLA-DR4 (DRA*0101 and DRB1*0401): insight into TCR cross-restriction and alloreactivity. J Exp Med 195: 571-581.
40. Buslepp J, Wang H, Biddison WE, Appella E, Collins EJ (2003) A correlation between TCR Valpha docking on MHC and CD8 dependence: implications for T cell selection. Immunity 19: 595-606.
41. Sethi DK, Schubert DA, Anders A-K, Heroux A, Bonsor DA, et al. (2011) A highly tilted binding mode by a self-reactive T cell receptor results in altered engagement of peptide and MHC. J Exp Med 208: 91-102.
42. Ishizuka J, Stewart-Jones GBE, van der Merwe A, Bell JI, McMichael AJ, et al. (2008) The structural dynamics and energetics of an immunodominant T cell receptor are programmed by its Vbeta domain. Immunity 28: 171-182.
43. Reiser J-B, Darnault C, Grégoire C, Mosser T, Mazza G, et al. (2003) CDR3 loop flexibility contributes to the degeneracy of TCR recognition. Nat Immunol 4: 241-247.
44. Reiser JB, Darnault C, Guimezanes A, Grégoire C, Mosser T, et al. (2000) Crystal structure of a T cell receptor bound to an allogeneic MHC molecule. Nat Immunol 1: 291-297.
45. Stewart-Jones GBE, McMichael AJ, Bell JI, Stuart DI, Jones EY (2003) A structural basis for immunodominant human T cell receptor recognition. Nat Immunol 4: 657-663.
46. Mazza C, Auphan-Anezin N, Gregoire C, Guimezanes A, Kellenberger C, et al. (2007) How much can a T-cell antigen receptor adapt to structurally distinct antigenic peptides? EMBO J 26: 1972-1983.
47. Saline M, Rödström KEJ, Fischer G, Orekhov VY, Karlsson BG, et al. (2010) The structure of superantigen complexed with TCR and MHC reveals novel insights into superantigenic T cell activation. Nat Commun 1: 119.
48. Reinherz EL, Tan K, Tang L, Kern P, Liu J, et al. (1999) The crystal structure of a T cell receptor in complex with peptide and MHC class II. Science 286: 1913-1921.
49. Reiser JB, Grégoire C, Darnault C, Mosser T, Guimezanes A, et al. (2002) A T cell receptor CDR3beta loop undergoes conformational changes of unprecedented magnitude upon binding to a peptide/MHC class I complex. Immunity 16: 345-354.

50. Wang J, Lim K, Smolyar A, Teng M, Liu J, et al. (1998) Atomic structure of an alphabeta T cell receptor (TCR) heterodimer in complex with an anti-TCR fab fragment derived from a mitogenic antibody. EMBO J 17: 10-26.
51. Hoare HL, Sullivan LC, Pietra G, Clements CS, Lee EJ, et al. (2006) Structural basis for a major histocompatibility complex class Ib-restricted T cell response. Nat Immunol 7: 256-264.
52. Yoshida K, Corper AL, Herro R, Jabri B, Wilson IA, et al. (2010) The diabetogenic mouse MHC class II molecule I-Ag7 is endowed with a switch that modulates TCR affinity. J Clin Invest 120: 1578-1590.
53. Kjer-Nielsen L, Clements CS, Purcell AW, Brooks AG, Whisstock JC, et al. (2003) A structural basis for the selection of dominant alphabeta T cell receptors in antiviral immunity. Immunity 18: 53-64.
54. Maynard J, Petersson K, Wilson DH, Adams EJ, Blondelle SE, et al. (2005) Structure of an autoimmune T cell receptor complexed with class II peptide-MHC: insights into MHC bias and antigen specificity. Immunity 22: 81-92.
55. Kjer-Nielsen L, Clements CS, Brooks AG, Purcell AW, McCluskey J, et al. (2002) The 1.5 A crystal structure of a highly selected antiviral T cell receptor provides evidence for a structural basis of immunodominance. Structure 10: 1521-1532.
