# Supplementary material for: Quantitative Analysis of the Association Angle between T-cell Receptor Vα/Vβ Domains Reveals Important Features for Epitope Recognition
Source: PLoS Comput Biol. 2015 Jul 17;11(7):e1004244. doi: 10.1371/journal.pcbi.1004244 (PMC4505886; doi:10.1371/journal.pcbi.1004244)
Supplement: S1 Fig — Pairwise Euler-angle distances (EAD) were determined for all MHC-bound TCR structures and the free TCR structures according to Formula 1. Structures containing more than one biological unit were merged to one unique geometry. The distance matrix was hierarchically clustered using the Ward update formula. For each subtree of the dendrogram, the au (approximately biased) and the bp (bootstrapping probability) according to the bootstrapping method [58] are provided. We identified six significant clusters of an au-value greater than 95%. The clusters are marked by colored boxes. (PDF) [file pcbi.1004244.s007.pdf]

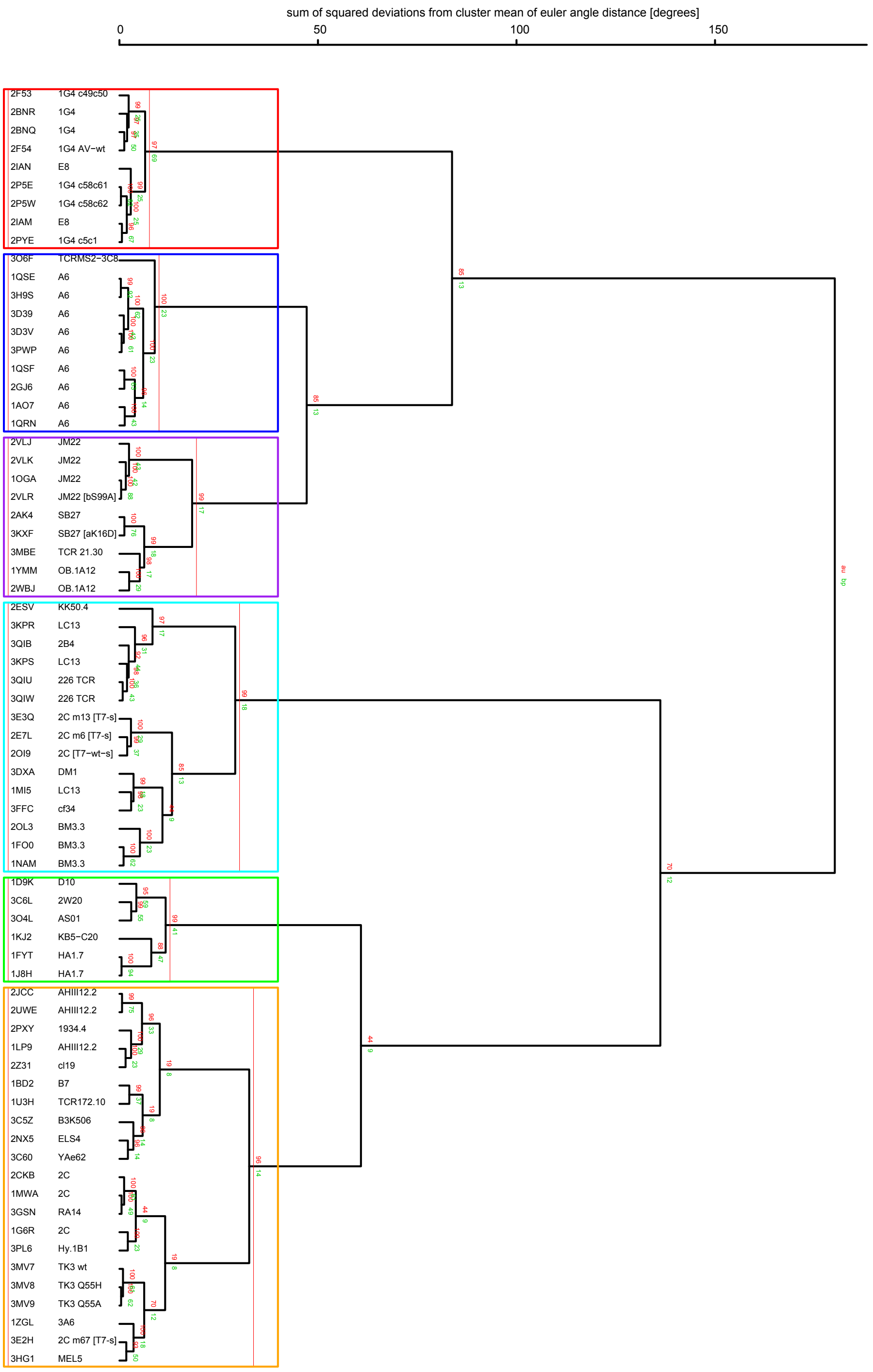

S1 Fig. Bootstrapping Dendrogramm of the Clustering of the MHC bound TCRs. Pairwise Euler-angle distances (EAD) were determined for all MHC-bound TCR structures and the free TCR structures according to Formula 1. Structures containing more than one biological unit were merged to one unique geometry. The distance matrix was hierarchically clustered using the Ward update formula. For each subtree of the dendrogram, the au (approximately biased) and the bp (bootstrapping probability) according to the bootstrapping method [1] are provided. We identified six siginificant clusters of an au-value greater than 95%. The clusters are marked by colored boxes.

1. Suzuki R, Shimodaira H (2006) Pvclust: an R package for assessing the uncertainty in hierarchical clustering. Bioinformatics 22: 1540-1542.
