# Supplementary material for: Quantitative Analysis of the Association Angle between T-cell Receptor Vα/Vβ Domains Reveals Important Features for Epitope Recognition
Source: PLoS Comput Biol. 2015 Jul 17;11(7):e1004244. doi: 10.1371/journal.pcbi.1004244 (PMC4505886; doi:10.1371/journal.pcbi.1004244)
Supplement: S2 Fig — For details see S1 Fig. The bootstrapping dendrogram was computed for the bound and free TCRs. The clusters of the unbound case are marked by colored boxes for comparison. Significant clusters are only found for smaller subtrees. (PDF) [file pcbi.1004244.s008.pdf]

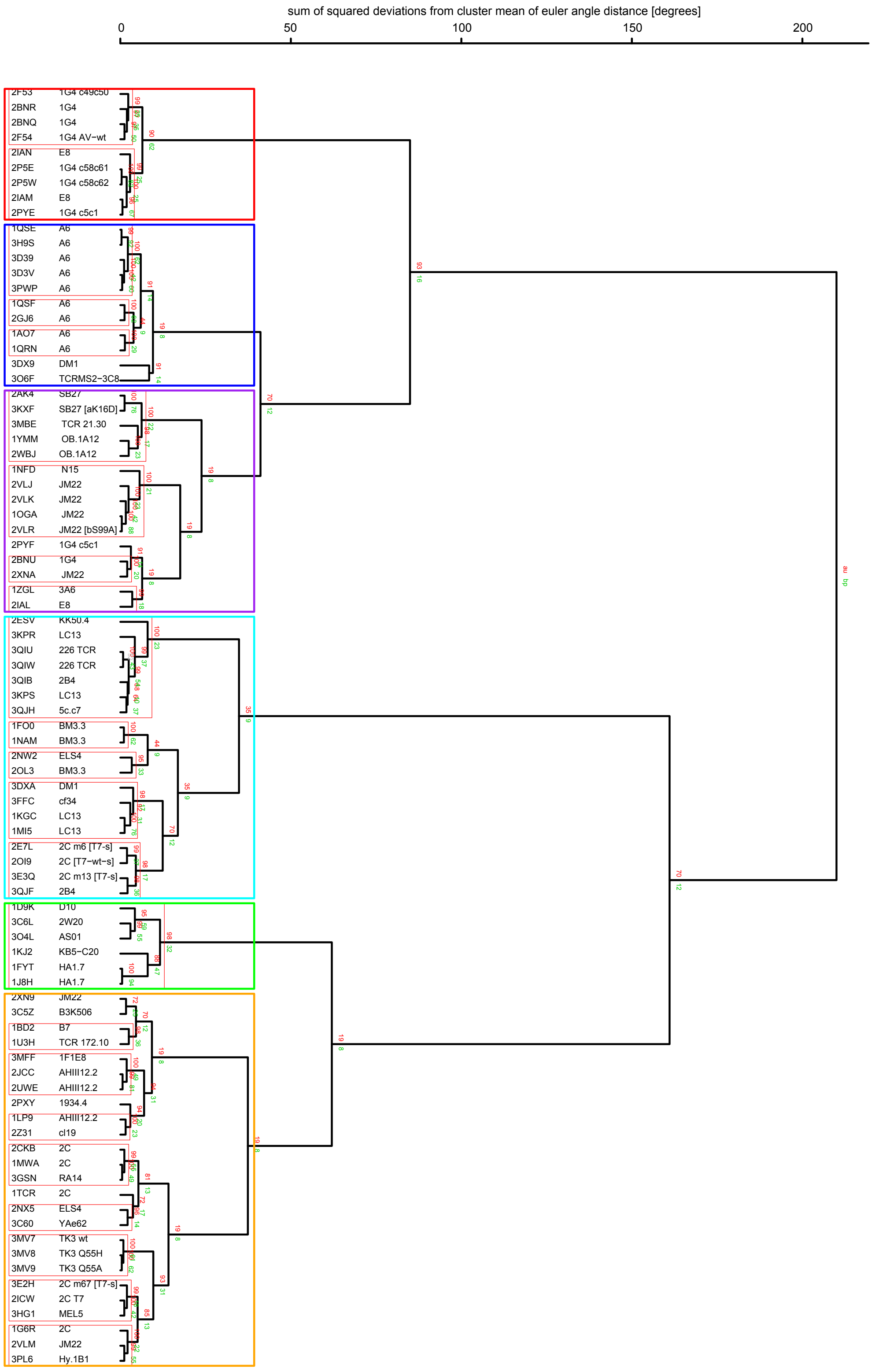

S2 Fig. Bootstrapping Dendrogram of the Clustering of the free TCRs together with the MHC bound TCRs. For details see S1 Fig. The bootstrapping dendrogram was computed for the bound and free TCRs. The clusters of the unbound case are marked by colored boxes for comparison. Significant clusters are only found for smaller subtrees.
