# Supplementary material for: Quantitative Analysis of the Association Angle between T-cell Receptor Vα/Vβ Domains Reveals Important Features for Epitope Recognition
Source: PLoS Comput Biol. 2015 Jul 17;11(7):e1004244. doi: 10.1371/journal.pcbi.1004244 (PMC4505886; doi:10.1371/journal.pcbi.1004244)
Supplement: S3 Fig — Pairwise Euler-angle distances (EAD) were determined for all MHC-bound TCR structures and the free TCR structures according to Formula 2 (see Methods). Structures containing more than one biological unit were merged to one unique geometry. The distance matrix was hierarchically clustered using the Ward update formula. We identified six significant clusters, using a bootstrapping approach [58]. Notably, in most of the cases, TCRs of the same type occur in the same cluster. Upper panel: Clustering dendrogram with bootstrapping results (au = approximately unbiased, bp = bootstrapping probability) Left panel: TCR types occurring within a cluster. Right/lower panel: Structure PDB identifiers and corresponding TCR names. MHC unbound TCR structures are indicated by bold-italics fonts. Central panel: Pairwise Euler-angle distances (EAD). The color key is provided in the bottom of the figure. (PDF) [file pcbi.1004244.s009.pdf]

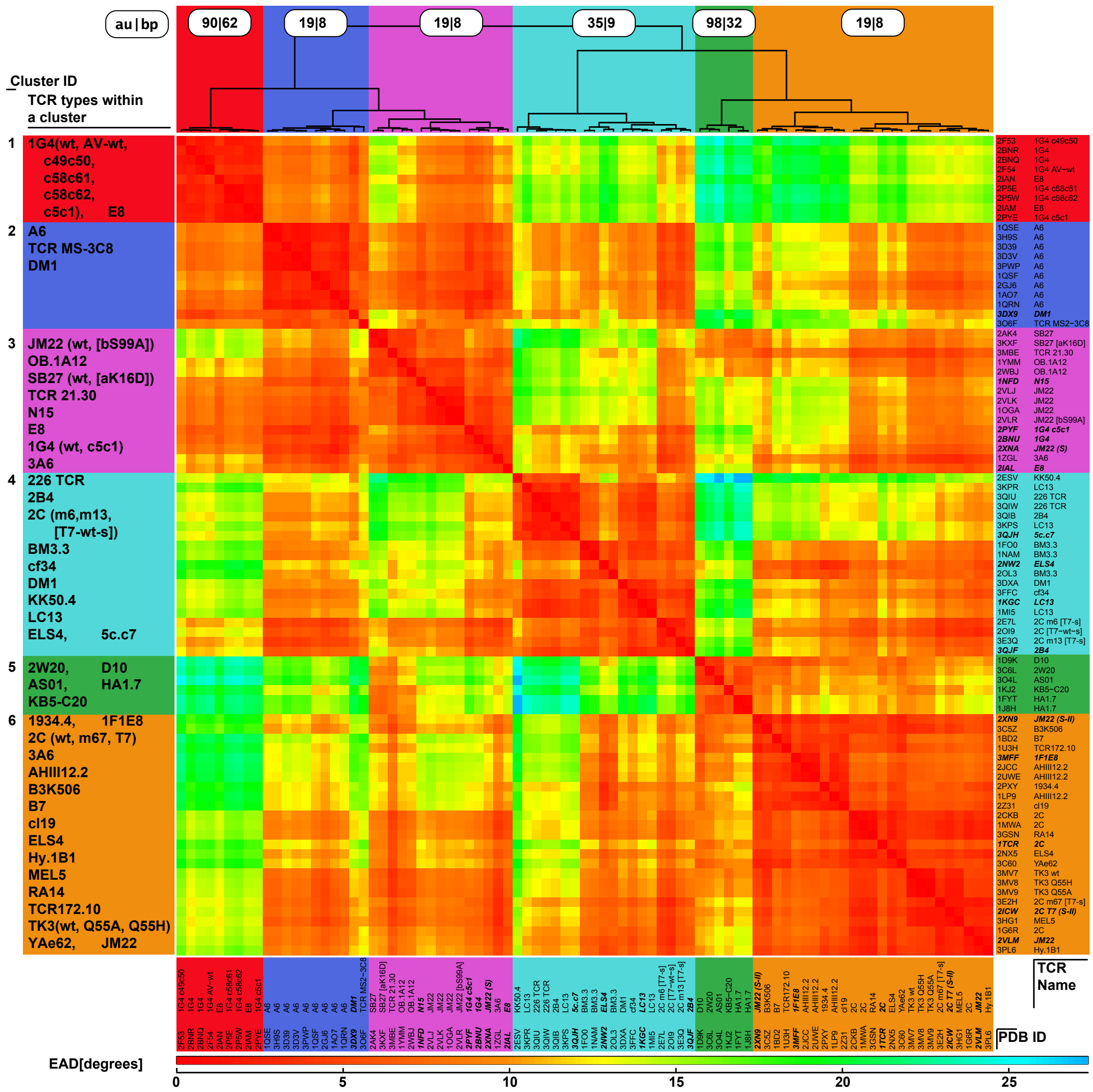

S3 Fig. Geometry Clusters of bound and unbound TCRs. Pairwise Euler-angle distances (EAD) were determined for all MHC-bound TCR structures and the free TCR structures according to Formula 2 (see Materials and Methods). Structures containing more than one biological unit were merged to one unique geometry. The distance matrix was hierarchically clustered using the Ward update formula. We identified six significant clusters, using a bootstrapping approach [1]. Notably, in most of the cases, TCRs of the same type occur in the same cluster. Upper panel: Clustering dendrogram with bootstrapping results (au=approximately unbiased, bp=bootstrapping probability) Left panel: TCR types occurring within a cluster. Right/lower panel: Structure PDB identifiers and corresponding TCR names. MHC unbound TCR structures are indicated by bold-italics fonts. Central panel: Pairwise Euler-angle distances (EAD). The color key is provided in the bottom of the figure.

1. Suzuki R, Shimodaira H (2006) Pvclust: an R package for assessing the uncertainty in hierarchical clustering. Bioinformatics 22: 1540-1542.
